# Morphometric and Molecular Interplay in Hypertension-Induced Cardiac Remodeling with an Emphasis on the Potential Therapeutic Implications

**DOI:** 10.3390/ijms26094022

**Published:** 2025-04-24

**Authors:** Lyubomir Gaydarski, Kristina Petrova, Stancho Stanchev, Dimitar Pelinkov, Alexandar Iliev, Iva N. Dimitrova, Vidin Kirkov, Boycho Landzhov, Nikola Stamenov

**Affiliations:** 1 Department of Anatomy, Histology and Embryology, Medical University of Sofia, 1431 Sofia, Bulgaria; kristinapetrova270@gmail.com (K.P.); stanchev_1989@abv.bg (S.S.); dpelinkovjr@gmail.com (D.P.); dralexiliev@abv.bg (A.I.); landzhov_medac@abv.bg (B.L.); nikola.stamenov1@gmail.com (N.S.); 2 Department of Cardiology, University Hospital “St. Ekaterina”, Medical University of Sofia, 1431 Sofia, Bulgaria; dimytrova@yahoo.com; 3 Department of Health Policy and Management, Faculty of Public Health ‘Prof. Dr. Tzekomir Vodenicharov’, Medical University of Sofia, 1527 Sofia, Bulgaria; vidinkk@abv.bg

**Keywords:** hypertensive myocardium, apelin, apelin receptor, vascular endothelial growth factor, nitric oxide synthase, fibrosis, capillary density, mast cells, therapeutic implications

## Abstract

Hypertension-induced cardiac remodeling is a complex process driven by interconnected molecular and cellular mechanisms that culminate in hypertensive myocardium, characterized by ventricular hypertrophy, fibrosis, impaired angiogenesis, and myocardial dysfunction. This review discusses the histomorphometric changes in capillary density, fibrosis, and mast cells in the hypertensive myocardium and delves into the roles of key regulatory systems, including the apelinergic system, vascular endothelial growth factor (VEGF)/VEGF receptor (VEGFR) pathways, and nitric oxide (NO)/nitric oxide synthase (NOS) signaling in the pathogenesis of hypertensive heart disease (HHD). Capillary rarefaction, a hallmark of HHD, contributes to myocardial ischemia and fibrosis, underscoring the importance of maintaining vascular integrity. Targeting capillary density (CD) through antihypertensive therapy or angiogenic interventions could significantly improve cardiac outcomes. Myocardial fibrosis, mediated by excessive collagen deposition and influenced by fibroblast growth factor-2 (FGF-2) and transforming growth factor-beta (TGF-β), plays a pivotal role in the structural remodeling of hypertensive myocardium. While renin–angiotensin–aldosterone system (RAAS) inhibitors show anti-fibrotic effects, more targeted therapies are needed to address fibrosis directly. Mast cells, though less studied in humans, emerge as critical regulators of cardiac remodeling through their release of pro-fibrotic mediators such as histamine, tryptase, and FGF-2. The apelinergic system emerges as a promising therapeutic target due to its vasodilatory, anti-fibrotic, and cardioprotective properties. The system counteracts the deleterious effects of the RAAS and has demonstrated efficacy in preclinical models of hypertension-induced cardiac damage. Despite its potential, human studies on apelin analogs remain limited, warranting further exploration to evaluate their clinical utility. VEGF signaling plays a dual role, facilitating angiogenesis and compensatory remodeling during the early stages of arterial hypertension (AH) but contributing to maladaptive changes when dysregulated. Modulating VEGF signaling through exercise or pharmacological interventions has shown promise in improving CD and mitigating hypertensive cardiac damage. However, VEGF inhibitors, commonly used in oncology, can exacerbate AH and endothelial dysfunction, highlighting the need for therapeutic caution. The NO/NOS pathway is essential for vascular homeostasis and the prevention of oxidative stress. Dysregulation of this pathway, particularly endothelial NOS (eNOS) uncoupling and inducible NOS (iNOS) overexpression, leads to endothelial dysfunction and nitrosative stress in hypertensive myocardium. Strategies to restore NO bioavailability, such as tetrahydrobiopterin (BH_4_) supplementation and antioxidants, hold potential for therapeutic application but require further validation. Future studies should adopt a multidisciplinary approach to integrate molecular insights with clinical applications, paving the way for more personalized and effective treatments for HHD. Addressing these challenges will not only enhance the understanding of hypertensive myocardium but also improve patient outcomes and quality of life.

## 1. Introduction

Hypertension, or high blood pressure, is a global public health challenge affecting approximately 1.13 billion individuals worldwide, with many remaining undiagnosed and untreated [1,2,3]. This condition is a leading risk factor for cardiovascular diseases, including heart failure (HF), coronary artery disease, and stroke, significantly contributing to morbidity and mortality [2,3,4,5]. The pathophysiology of arterial hypertension (AH) involves complex interactions among genetic, environmental, and lifestyle factors that result in vascular remodeling, increased resistance, and heightened cardiac workload [3,6]. Chronic pressure overload induces structural and functional changes in the cardiovascular system, manifesting as left ventricular hypertrophy (LVH), myocardial fibrosis, and diastolic dysfunction, collectively termed hypertensive cardiomyopathy, which progresses to HF if untreated [5,6].

Hypertensive heart disease (HHD) is a major consequence of chronic AH, characterized by LVH, fibrosis, and impaired myocardial relaxation, often leading to HF and other complications such as arrhythmias and coronary artery disease [5,7,8]. The remodeling of the hypertensive myocardium is mediated by molecular mechanisms, including renin–angiotensin–aldosterone system (RAAS) activation, oxidative stress, and inflammatory pathways, with key roles played by pro-inflammatory cytokines and growth factors such as endothelin-1 and transforming growth factor-beta [5,8,9,10,11,12]. These mechanisms culminate in myocardial stiffness, fibrosis, and diastolic dysfunction, ultimately contributing to HF [5].

Several morphological markers are used to assess myocardial remodeling in hypertensive conditions. These include cardiac muscle hypertrophy, measured by the thickness of the heart wall [13,14], the cross-sectional area of cardiomyocytes [15,16], capillary density (CD) [15,17,18,19], and fibrosis [20,21]. Healthy myocardial CD, typically ranging from 2900 to 4000 capillaries per mm^2^, ensures adequate oxygenation and nutrient delivery to cardiomyocytes [22]. However, AH diminishes CD, exacerbating oxygen deprivation and promoting myocardial ischemia. Concurrently, fibrosis is characterized by excessive accumulation of extracellular matrix (ECM) components, and more specifically collagen [21,23]. The main type of collagen found in healthy hearts is type I, accounting for around 85% of the total amount, followed by type III, representing around 11%. Two main types of fibrosis are described in the heart—reparative and reactive [20]. In the first one, fibrosis is developed at the sites of cardiomyocytes necrosis, while the second type is based on diffuse collagen deposition throughout the myocardium without connection to cell death [24]. Mast cells are sedentary, mononuclear cells originating from the myeloid lineage in the bone marrow [19,25,26]. Two different subtypes of mast cells are described in the literature—connective tissue mast cells (CTMCs) and mucosal mast cells [27,28]. Of those two subtypes, CTMCs found in the myocardium participate in numerous pathological pathways and ultimately in myocardial remodeling [28,29]. Moreover, mast cells emerge as critical regulators in the hypertensive myocardium, linking capillary density, fibrosis, and angiogenesis. Through their extensive repertoire of bioactive substances, including vascular endothelial growth factor (VEGF) and fibroblast growth factor-2 (FGF-2), mast cells not only promote angiogenesis but also contribute to inflammatory and fibrotic processes [30,31].

Current AH treatments focus on lowering blood pressure and reducing associated risks by targeting different physiological pathways. Classical antihypertensive treatments consist of modulators of the RAAS system and/or diuretics. Two key classes, mineralocorticoid receptor blockers and sodium–glucose cotransporter-2 inhibitors, offer distinct benefits [32]. The current approach to optimizing antihypertensive treatment is shifting towards personalized strategies, with an emphasis on targeting novel molecular and enzymatic systems that could serve as promising avenues for improved therapy [33,34,35]. A potentially promising therapeutic target could be the apelinergic system, the VEGF/VEGF receptor (VEGFR) pathway, nitric oxide (NO)/nitric oxide synthase (NOS) signaling, as well as factors related to fibrosis, capillary density, and mast cells.

The apelinergic system, comprising the apelin receptor (APLNR) and its ligands apelin and elabela, plays a critical role in cardiovascular regulation, acting through mechanisms that impact vasodilation, angiogenesis, and blood pressure control. Apelin, synthesized as a 77-amino-acid pre-pro-peptide, is processed into active isoforms, with pyr-apelin 13 being the most abundant in the human heart due to its high binding affinity for APLNR [36,37,38]. APLNR is expressed predominantly in vascular endothelial cells and, to a lesser extent, in vascular smooth muscle cells, enabling it to mediate vasodilation and antagonize RAAS. This antagonism is achieved through receptor dimerization, which reduces the affinity of angiotensin II (ANG II) for its type 1 receptor, thereby preserving cardiovascular homeostasis [39,40]. Moreover, while Angiotensin II (Ang II) promotes vasoconstriction and fibrosis, apelin typically induces vasodilation and exhibits anti-fibrotic properties [41]. This contrasting functionality immediately suggests a potential counter-regulatory relationship, where dysfunction in apelin signaling could exacerbate pathological processes driven by Ang II in hypertension, a key factor in HHD development. Apelin also stimulates angiogenesis via NO-dependent pathways, activating AMPK and Akt signaling [42,43,44]. These endothelial-dependent effects highlight the critical role of the apelinergic system in maintaining vascular integrity and reducing cardiac preload and afterload [45,46,47,48,49]. Given the profound impact of HHD and the multifaceted roles of the apelinergic system in cardiovascular physiology, understanding the interplay between this system and the pathophysiology of HHD is of considerable scientific and clinical interest.

Notably, APLNR activation is closely linked to VEGF signaling, further enhancing its role in angiogenesis. VEGF-A expression, stimulated by APLNR activation, promotes endothelial cell proliferation and vascular permeability, essential for capillary formation and myocardial angiogenesis [49,50]. VEGF-A, predominantly VEGF-A165, is the most potent isoform, facilitating localized signaling through heparin proteoglycans [51,52,53,54]. VEGF signaling operates through VEGFR1, VEGFR2, and VEGFR3, with VEGFR2 serving as the principal mediator of angiogenic signaling in the myocardium, critical for cardiomyocyte survival and adaptive hypertrophy [55,56,57]. Dysregulated VEGF pathways can lead to impaired angiogenesis and contribute to myocardial ischemia, underscoring the therapeutic potential of targeting VEGF receptors in cardiovascular diseases.

The NO signaling system, regulated by NOSs, further integrates with these pathways to modulate cardiovascular function. Endothelial NOS (eNOS) and neuronal NOS (nNOS) play central roles in NO production, which facilitates vasodilation, angiogenesis, and myocardial relaxation [58]. While eNOS is predominantly localized in caveolae, regulating mitochondrial respiration and negative inotropic effects, nNOS is associated with the sarcoplasmic reticulum and mitochondria, influencing calcium handling and cardiac rhythm [59,60]. Importantly, NO pathways overlap with VEGF signaling, as VEGF-induced angiogenesis is partly mediated by NO production via the Akt/eNOS pathway [61,62].

This intricate interplay between capillary density, fibrosis, mast cells, the apelinergic system, VEGF/VEGFR signaling, and the NO/NOS pathways underscores the complexity of cardiovascular regulation. These interconnections reveal potential therapeutic opportunities for mitigating hypertensive damage and enhancing myocardial health through the targeted modulation of these pathways. This review aims to assess the role of these targeted molecular pathways and their interplay with the morphometric markers of myocardial remodeling, to compare findings from animal and human studies, and to evaluate their potential future therapeutic implications.

## 2. Assessment of Myocardial Histomorphometric Parameters and Their Role in Myocardial Remodeling in the Context of AH

### 2.1. Role of Capillary Density (CD) in the Context of AH and Hypertensive Myocardium

#### 2.1.1. Animal Studies

Studies investigating CD in spontaneously hypertensive rats (SHRs) present conflicting findings. Some studies report an increase in CD in SHRs compared to normotensive Wistar–Kyoto (WKY) rats, suggesting a compensatory mechanism to counteract worsening hypoxia [63]. This increase has been associated with elevated VEGF expression and a higher capillary-to-fiber ratio in the SHRs heart [63]. Olianti et al. also observed an increase in CD in both the left (LV) and right ventricles (RV) of SHRs, with these findings spanning multiple age groups, including 4-week-old rats [64]. Since AH in SHRs typically develops between 5 and 6 weeks of age, this suggests that the observed increase in CD occurs even earlier, indicating that vascular remodeling begins before significant changes in blood pressure are evident [64,65].

Conversely, other studies present contradictory evidence. Caudron et al. reported a decrease in CD in SHRs, particularly in older animals with more severe hypertension-induced cardiac injury [66]. Similar capillary rarefaction has also been documented in other hypertensive animal models, such as Dahl salt-sensitive rats and C57BL/6J mice subjected to a high-salt diet combined with ANG II [67,68,69]. Stanchev et al. propose that this capillary rarefaction is linked to reduced VEGF expression in the SHRs heart and plays a significant role in hypertension-induced cardiac injury [70].

#### 2.1.2. Human Studies

Data on CD in human hearts are relatively scarce, and conclusions are often drawn indirectly. Cheng et al. analyzed CD in 150 adults aged 19 to 55, divided into three groups: normotensive, untreated hypertensive, and hypertensive under therapy. CD was assessed in nailfold skin and showed no significant differences in structural CD but revealed increased functional capillary rarefaction [71]. These findings align with those of Prewitt et al., who described an initial functional capillary rarefaction, followed by irreversible structural capillary rarefaction [72].

Contrary to these results, Penna et al. reported an approximately 25% structural capillary deficit in hypertensive patients compared to normotensive individuals [73]. A more recent study using biopsy samples from patients with HHD revealed a CD of 1162 ± 189 mm^2^, significantly lower than the 2249 ± 85 mm^2^ observed in healthy normotensive hearts [74]. This pronounced capillary rarefaction in hypertensive individuals underscores the critical role of the vascular network in maintaining proper heart function. Further research in this area could provide deeper insights into the pathomorphological and pathophysiological mechanisms underlying hypertension-induced cardiac injury. The discussed animal and human studies on CD are summarized in Table 1.

#### 2.1.3. Potential Therapeutic Implications

Capillary rarefaction plays a critical role in the pathological changes associated with HHD. It contributes to hypoxia and cell death while promoting fibrosis [75,76]. As a result, assessing the capillary network is valuable for staging and prognosticating the progression of hypertensive cardiac changes, such as hypertrophy, and for evaluating the risk of more severe complications, including HF.

Some studies have shown improved CD in patients undergoing chronic antihypertensive therapy, suggesting that the morphological evaluation of capillaries could serve as an indicator of treatment efficacy [77]. Another study explored strategies to actively influence capillary density. Data from Van Kerckhoven et al. demonstrated that post-infarction patients treated with methylprednisolone, moxonidine, and captopril exhibited improved capillary density. Furthermore, their findings on aspirin use indicated even more favorable outcomes, suggesting enhanced neovascularization in the spared myocardial tissue following infarction [78].

### 2.2. Role of Fibrosis (FGF, FGFR/Collagen Types) in the Context of AH and Hypertensive Myocardium

#### 2.2.1. Animal Studies

Hypertension-induced heart fibrosis in SHRs is predominantly of the reactive type. Experiments examining the LV and RV of SHRs reveal increased wall thickness and elevated collagen deposition in the cardiac interstitium compared to normotensive WKY rats [15,16,79]. These alterations are more pronounced in the LV than in the RV, primarily due to the greater mechanical stress on the left side of the heart [19,80]. Furthermore, the extent of interstitial fibrosis is significantly higher in 12-month-old SHRs, representing advanced HHD, compared to 6-month-old SHRs, in which AH is still developing [19].

The fibrotic changes result from excessive deposition of collagen types I and III, coupled with normal or downregulated collagen degradation by matrix metalloproteinases [81]. One of the main cell-signaling pathways implicated in collagen upregulation and subsequent fibrosis involves FGF-2 [19,82]. Data from SHRs indicate a moderate increase in FGF-2 expression in the LV and RV of 12-month-old rats compared to 6-month-old rats. This finding correlates with regions of more pronounced fibrotic changes and higher FGF-2 levels [19].

Another study focused on SHRs demonstrates elevated procollagen type I messenger RNA expression in the LV [83,84]. Additionally, it describes reduced collagenase activity, which disrupts the balance between collagen deposition and degradation [83,84]. The role of collagen deposition in the pathogenesis and progression of HHD is summarized in Table 2.

Another key pathway in cardiac remodeling involves ANG II and its profibrotic and proinflammatory effects. Komaki et al. report a significant increase in blood pressure in SHRs following ANG II administration, accompanied by increased interstitial myocardial fibrosis. They also describe a link between elevated ANG II levels and transforming growth factor-beta (TGF-β) [85]. TGF-β, a multifunctional cytokine, plays a critical role in cardiac remodeling by promoting the transformation of fibroblasts into myofibroblasts and enhancing the profibrotic activity of endothelial cells [86]. Table 3 is a summary of the key drivers of fibrosis in the pathogenesis of HHD.

#### 2.2.2. Human Studies

In humans, the composition of the ECM is similar to that observed in SHRs. Collagen type I remains the predominant type, followed by collagen type III [87]. Histologically, four distinct types of fibrotic changes can be identified: interstitial, compact, diffuse, and patchy [88]. In all four types, the primary alteration is the increased deposition of collagen types I and III [88]. This increase is driven by the transformation of fibroblasts into myofibroblasts, which subsequently produce ECM components, including collagen [89].

It is important to note that fibrotic changes in the heart are closely linked with inflammation, which can further exacerbate elevated collagen deposition through higher rates of myofibroblast transformation and activation [90]. At sites of inflammation, the influx of immunocompetent cells leads to increased levels of TGF-β, a potent factor for activating fibroblasts into myofibroblasts [91,92]. Almendral et al. reported elevated serum levels of TGF-β in hypertensive patients, which correlated with increased left ventricular (LV) wall thickness, further supporting the role of TGF-β in cardiac fibrosis [93].

Similar to the findings in animal models, humans with AH exhibit elevated levels of ANG II [94]. Studies using ANG II blockade have demonstrated reduced fibrotic changes in the heart, highlighting its potential therapeutic role [95,96]. Table 4 summarizes and compares the core findings in animal and human studies on fibrosis.

#### 2.2.3. Potential Therapeutic Implications

Although the heart is one of the organs most affected in patients with AH, no antifibrotic medications have been approved for the treatment of cardiovascular diseases [97]. Among the most commonly prescribed drugs for patients with HHD are RAAS inhibitors. These drugs not only lower blood pressure but also reduce ECM expansion in the myocardium [98]. This effect may be partially attributed to the anti-TGF-β activity of certain RAAS inhibitors, such as losartan [99].

Inflammation modulators are also showing promising results in experimental studies. Colchicine has been demonstrated to reduce the extent of fibrotic changes in animal models of HF or following myocardial infarction [98,100,101].

Inhibiting MAPK signaling, particularly targeting the p38 MAPK pathway, has demonstrated anti-fibrotic effects in preclinical models [102]. However, caution is needed because p38α also plays a role in maintaining scar integrity, making it a potentially double-edged therapeutic target [102]. Another emerging strategy involves modulation of calcium signaling, specifically by targeting calcium channels such as TRPC and Orai1, or downstream effectors like calcineurin, which are involved in fibroblast activation and cardiac hypertrophy [103].

Inhibiting collagen cross-linking is also being explored as a therapeutic approach. Lysyl oxidase (LOX) enzymes, which contribute to excessive collagen cross-linking and increased myocardial stiffness, are a potential target [104]. However, developing selective inhibitors for specific LOX isoforms remains a significant challenge [105].

Targeting the FGF system offers multiple opportunities due to its complex and context-dependent roles. FGF21 agonism, using recombinant proteins or analogs, is currently under investigation for its protective metabolic and cardiovascular effects [106]. On the other hand, antagonizing FGF23 or inhibiting FGFR4 may block the pro-hypertrophic effects of excess FGF23, especially relevant in conditions like chronic kidney disease (CKD) and HHD [107]. FGF2 also has potential anti-fibrotic effects, but its signaling is highly context-dependent and isoform-specific, with some forms also promoting hypertrophy. Therefore, a more refined understanding of FGF2 biology is necessary before it can be effectively targeted [108].

Anti-inflammatory strategies, such as targeting monocyte chemoattractant protein-1 (MCP-1) or modulating macrophage activity, could help mitigate fibrosis linked to chronic inflammation in HHD [109]. However, the complexity and redundancy of these signaling networks make the development of effective therapies challenging. For example, pathways like TGF-β/SMAD3 are essential for tissue repair, meaning their inhibition may have unintended consequences. Similarly, molecules like FGF2 can have both beneficial and detrimental effects depending on the context.

To overcome these challenges, future therapies may need to be highly specific—targeting particular receptor isoforms (e.g., FGFR4), downstream signaling nodes, or using combination therapies that simultaneously address multiple pathways. Personalized approaches tailored to individual patient characteristics, such as the presence of CKD or the type of heart failure (HFpEF vs. HFrEF), may also be necessary. Continued research into the molecular mechanisms of fibrosis, collagen regulation, and FGF signaling in the hypertensive heart is crucial for translating these insights into successful clinical treatments.

### 2.3. Role of Mast Cells in the Context of AH and Hypertensive Myocardium

#### 2.3.1. Animal Studies

Kotov et al. reported a statistically significant increase in mast cell numbers (MCNs) between 6-month-old and 12-month-old SHRs. Their data also revealed a strong correlation between MCN, FGF-2 expression levels, and increased fibrotic areas [19]. As previously discussed, FGF-2 plays a key role in cardiac fibrosis, and since mast cells store and release FGF-2, they represent an important source of this profibrotic agent, particularly when their numbers are elevated in HHD. Notably, the observed increase in MCN was present in both the LV and RV, though it was less pronounced in the RV [19].

In addition to FGF-2, mast cells possess an extensive neurochemical profile, including tryptase, chymase, and histamine, all of which have profibrotic roles and contribute to fibroblast activation and subsequent myofibroblast transformation [29,110,111]. McLarty et al. identified a specific pathway for fibroblast activation mediated by tryptase. Tryptase stimulates protease-activated receptor 2 (PAR-2), triggering mitogen-activated protein kinase (MAPK) activation and phosphorylation of extracellular signal-regulated kinase isoforms 1 and 2 (ERK 1/2). This ultimately drives fibroblast transformation into myofibroblasts, leading to increased collagen synthesis [112]. Similarly, Tan et al. reported that tryptase significantly increases cell proliferation and collagen I synthesis in Sprague Dawley rats [113]. Chymase also exhibits profibrotic effects, including the activation of TGF-β, which potentially links it to the previously discussed increase in cardiac fibrosis [114]. Additionally, chymase has been shown to generate ANG II [115], which provides another pathway for it to induce proinflammatory and profibrotic changes. Regarding histamine, studies have revealed elevated histamine levels and increased histamine-2-receptor (H2R) expression in SHRs compared to normotensive Wistar rats [116]. This aligns with the increased MCN in SHRs, as mast cells are the primary source of histamine in the heart. It is worth noting that, while most studies emphasize the profibrotic properties of mast cells, Legere et al. reported that mast cells release both antifibrotic and profibrotic mediators depending on microenvironmental signals [29]. This highlights the nuanced and complex role of mast cells in cardiac remodeling. Key cardiac mast cell mediators and their implicated roles in HHD pathophysiology are summarized in Table 5.

#### 2.3.2. Human Studies

Mast cell numbers (MCNs) are reported to increase in patients with dilated cardiomyopathy [117]. Another study focused on transplanted human hearts demonstrated a correlation between cardiac mast cells and interstitial and perimyocytic fibrosis [118]. However, there is a lack of human studies specifically investigating MCN and their role in the development of cardiac remodeling under hypertensive conditions.

Currently, the only way to evaluate the role of mast cells in humans is through their neurochemical profile. FGF-2, tryptase, chymase, and histamine in humans exhibit the same profibrotic effects described in animal models. These include fibroblast activation and myofibroblast transformation, promotion of inflammation, and ANG II generation [119,120,121]. However, no studies have directly connected MCN with increased expression of these neurochemicals in the context of HHD in humans.

Animal studies strongly suggest that mast cells play a key role in cardiac remodeling. Further human experiments are necessary to clarify their role in cardiac fibrosis and potentially uncover novel therapeutic approaches. Table 6 provides thorough comparison of the role of mast cells in HHD progression in animals and humans.

#### 2.3.3. Potential Therapeutic Implications

Drugs targeting mast cell activation are currently undergoing clinical trials for mast cell activation disorders [29,122]. However, their potential role in HHD and cardiac remodeling remains unexplored. Additionally, novel drugs that inhibit IgE-FcɛRI interactions on mast cells are showing promising results in addressing cardiac fibrosis and remodeling [123].

Another promising approach to mitigate mast cell effects involves the use of chymase inhibitors, which may reduce proinflammatory responses and limit ANG II formation [120]. Muhammad et al. are exploring tryptase inhibitors as potential anti-inflammatory agents, although these studies are not specifically focused on cardiac anti-fibrotic applications [124]. Importantly, chymase inhibitors have also been shown to reduce histamine release, which could contribute to an anti-fibrotic effect in the heart [124].

Tryptase inhibitors represent a potential anti-fibrotic strategy, as tryptase is known to activate fibroblasts and contribute to tissue fibrosis [125]. In addition to its fibrotic role, elevated serum tryptase levels have been associated with greater severity of coronary artery disease and plaque instability in some human studies, suggesting that tryptase might serve as a biomarker for cardiovascular risk. However, its specific utility in HHD remains to be investigated [126].

Histamine receptor antagonists, particularly H2 receptor blockers, have also emerged as a possible therapeutic option. A large cohort study found that the use of H2 receptor antagonists was associated with a lower risk of developing heart failure, which is a noteworthy observation [127]. However, this finding needs to be confirmed in prospective, randomized clinical trials before H2 blockers can be recommended for heart failure prevention or treatment based on their interaction with histamine signaling.

Anti-IgE therapy, such as omalizumab, is another emerging area of interest. Recent studies have identified a novel IgE–mast cell (MC)–IL-6–endothelial dysfunction pathway in the pathophysiology of hypertension, providing a rationale for investigating anti-IgE interventions [128]. In experimental models, omalizumab—a monoclonal antibody targeting IgE—attenuated angiotensin-II-induced hypertension and vascular remodeling in mice [129]. Although omalizumab is currently approved for treating allergic asthma and chronic urticaria, its cardiovascular effects in humans remain largely unknown. This highlights the need for dedicated studies in patient populations with cardiovascular comorbidities.

Finally, anti-cytokine therapies targeting pro-inflammatory and pro-fibrotic cytokines released by cardiac mast cells—such as IL-6 (implicated via the IgE pathway) [128], TNF-α, or TGF-β—could represent another potential therapeutic approach. However, achieving specificity in targeting these cytokines remains a significant challenge. Table 7 represents a short summary of mast-cell-targeted therapeutic strategies.

**Figure 1:**
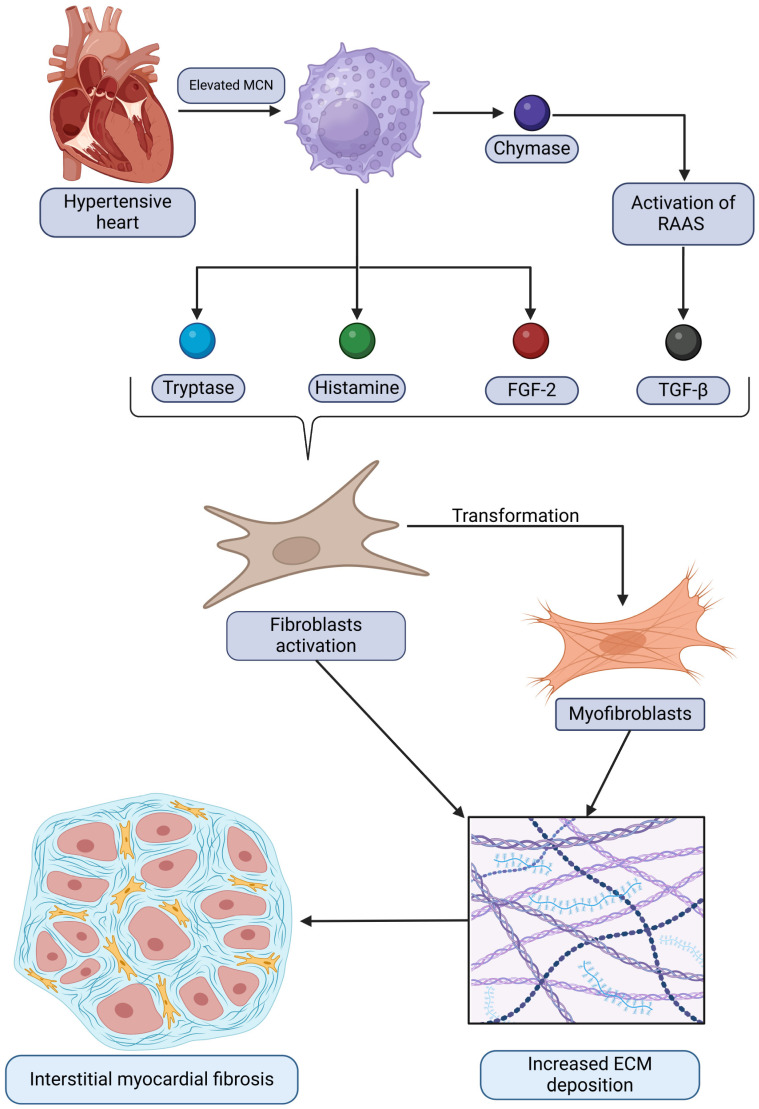
A graphical representation of the relationship between hypertensive heart disease and elevated mast cell numbers (MCNs), which subsequently drive the increased expression of certain bioactive substances. This process activates fibroblasts and triggers their transformation into myofibroblasts. Ultimately, these changes result in augmented extracellular matrix (ECM) deposition and the development of interstitial myocardial fibrosis. Original figure created with BioRender.com.

The relationships between mast cells and the development of interstitial myocardiac fibrosis in the context of HHD discussed are summarized and demonstrated in Figure 1. It depicts how mast cells contribute to pathological remodeling by releasing mediators that activate fibroblasts and promote extracellular matrix deposition, ultimately leading to fibrosis.

## 3. Assessment of the Molecular Modalities in AH and Their Role for Myocardial Remodeling

### 3.1. Role of the Apelinergic System in the Context of AH and Hypertensive Myocardium

The apelinergic system is a complex signaling network comprising peptide ligands and their cognate receptor, playing significant roles in diverse physiological processes, particularly within the cardiovascular system. Two distinct families of endogenous peptide ligands activate the APLNR receptor: apelin and Elabela peptides [130].

Apelin: The primary ligand, which originates from the APLN gene located on the X chromosome (Xq25–26.1 in humans) [130]. This gene encodes a 77-amino acid prepropeptide [130]. Post-translational processing involves cleavage of an N-terminal signal peptide, yielding a 55-amino acid precursor, pro-apelin-55. Pro-apelin-55 undergoes further proteolytic cleavage at specific di-basic amino acid motifs, likely by proprotein convertases, generating a family of shorter, biologically active C-terminal fragments [131]. The major identified isoforms include apelin-36 (residues 42–77), apelin-19, apelin-17 (61–77), apelin-13 (65–77), and apelin-12. The C-terminal 17 amino acids are highly conserved across species (bovine, human, rat, mouse), suggesting functional importance. Notably, the biological activity of these isoforms appears inversely proportional to their length, with apelin-13 generally considered the most potent, followed by apelin-17 and then apelin-36 [130]. Apelin-13 can undergo further modification at its N-terminal glutamine residue through pyroglutamation, catalyzed by glutamine cyclase, forming [Pyr1]apelin-13. This modification confers resistance to degradation by exopeptidases, resulting in a longer biological half-life and enhanced activity, making it a commonly used form in research and considered a key physiological ligand [130]. While the precise processing enzymes are not fully elucidated, angiotensin-converting enzyme 2 (ACE2) and pro-protein convertase subtilisin/kexin type 9 (PCSK9) have been implicated, with ACE2 potentially degrading certain apelin isoforms and PCSK9 possibly cleaving pro-apelin-55 directly to apelin-13 [130]. Pre(pro)apelin may exist as a dimer mediated by disulfide bonds within the signal peptide, potentially influencing processing, although the bioactive forms are monomeric [131].

Elabela (ELA/Toddler/Apela): More recently, a second endogenous ligand for APLNR, Elabela (ELA), was discovered [131]. Encoded by the APELA gene, ELA is structurally unrelated to apelin peptides. It exists in multiple isoforms, such as ELA-32, ELA-21, and ELA-11. ELA plays a critical role during embryonic development, particularly in cardiovascular morphogenesis, including heart formation and vasculogenesis [132]. Its discovery helped explain phenotypic discrepancies between apelin-knockout and APLNR-knockout mice, as APLNR deficiency causes severe developmental defects not seen with apelin deficiency alone, implying ELA is the crucial ligand during early development [132]. Importantly, ELA and its receptor are also expressed and functionally active in the adult cardiovascular system, suggesting ongoing roles beyond development [131].

The existence of two distinct ligand families (apelin and ELA) activating the same receptor introduces significant complexity. It implies that APLNR signaling can be initiated by different stimuli under potentially different physiological or developmental contexts. This suggests potentially distinct, overlapping, or temporally separated roles for apelin and ELA in cardiovascular regulation and disease, meaning research focused solely on apelin might provide an incomplete picture of the apelinergic system’s function.

The APLNR, encoded on human chromosome 11q12.1), is the central component through which both apelin and ELA exert their effects [133]. It was first identified in 1993 as an orphan receptor due to its sequence homology (31–54% identity, particularly in transmembrane regions) to the Angiotensin II Type 1 Receptor (AT1R). However, despite this similarity, APLNR does not bind Ang II or related peptides [133]. It remained an orphan receptor until apelin was identified as its endogenous ligand in 1998 [130]. APLNR is a class A (Rhodopsin-like) G protein-coupled receptor (GPCR) comprising 377–380 amino acids, possessing the characteristic seven transmembrane α-helical segments [133]. Its structure includes consensus sites for post-translational modifications like phosphorylation, palmitoylation, and glycosylation. Recent crystallographic studies, including structures of APLNR complexed with apelin-17 mimetics, have provided significant insights into its ligand-binding mode [133].

Regulation of APLNR expression is complex and not fully characterized. Transcriptional control involves promoter regions influenced by transcription factors like Sp1 (a major regulator), estrogen and glucocorticoid receptors, and CCAAT enhancer-binding protein γ (C/EBPγ). Physiological stimuli such as stress, salt loading, and water deprivation can induce APLNR synthesis. Hypoxia, via HIF-1α, also induces APLNR expression [134]. There is some evidence suggesting that apelin itself might regulate APLNR expression in certain tissues [135]. Several single-nucleotide polymorphisms (SNPs) in the APLNR gene have been identified and linked to cardiovascular phenotypes, including slower heart failure progression in idiopathic dilated cardiomyopathy and potential associations with hypertension or coronary artery disease [132].

The apelin/APLNR system exhibits a remarkably widespread distribution throughout the body, consistent with its diverse physiological roles [133]. Expression is prominent in the central nervous system (CNS), including the hypothalamus, pituitary gland, cerebral cortex, and hippocampus, implicating it in neuroendocrine functions, fluid homeostasis, and stress responses [136]. Peripherally, the system is found in the lungs, kidneys, gastrointestinal tract (stomach, intestine), adipose tissue, placenta, immune cells (T-lymphocytes), pancreatic islets, and platelets [133].

Within the cardiovascular system, both apelin and APLNR are highly expressed, underscoring their importance in cardiac and vascular function [132]. High levels of apelin mRNA and APLNR binding sites are detected in the heart [136]. Specifically, APLNR receptors are located on cardiomyocytes, endocardial cells, and vascular smooth muscle cells (VSMCs) [132]. Apelin expression is primarily localized to endothelial cells of various vessels, including small intramyocardial, renal, pulmonary, and adrenal vessels, coronary arteries, large conduit vessels, and endocardial endothelial cells [136]. This distribution pattern, with ligand production mainly in the endothelium and receptor expression on adjacent cardiomyocytes and VSMCs, strongly supports a paracrine mode of signaling within the heart and vasculature [137]. Elabela expression has also been confirmed in adult human heart and vascular endothelium [131].

Upon binding of apelin or Elabela, the APLNR receptor activates intracellular signaling pathways characteristic of G protein-coupled receptors (GPCRs), leading to diverse cellular responses. G protein coupling: The primary signaling mechanism involves coupling to inhibitory G proteins of the Gαi/o family [131]. Activation of Gαi/o typically leads to the inhibition of adenylyl cyclase, resulting in decreased intracellular cyclic AMP (cAMP) levels [131]. This inhibitory coupling is sensitive to pertussis toxin [138]. Elabela also activates APLNR through G protein-dependent pathways [131]. 

Downstream pathways: Activation of APLNR triggers several key downstream signaling cascades: PI3K/Akt pathway—the phosphatidylinositol 3-kinase (PI3K)/Akt pathway is frequently activated by apelin/APLNR signaling [131]. This pathway is crucial for cell survival, proliferation, and metabolism, e.g., enhancing glucose uptake [139], and it plays important roles in angiogenesis [140] and anti-apoptotic effects [141]. Interestingly, the role of PI3K/Akt may be context-dependent; for instance, while often protective, Apelin-13 was reported to inhibit this pathway in Ang II-stimulated cardiac fibroblasts—contributing to its anti-fibrotic effect in that specific setting, yet it can also activate PI3K/Akt to promote cardiomyocyte hypertrophy via autophagy in another study [142]. ELA may also activate PI3K/Akt signaling [143]. 

The extracellular signal-regulated kinase 1/2 (ERK1/2) pathway, as part of the mitogen-activated protein kinase (MAPK) cascade, is another common target. ERK activation is implicated in cell growth, proliferation, survival, angiogenesis, and the positive inotropic effects of both apelin and Elabela [131]. However, its role in hypertrophy is complex, as one study showed that apelin inhibited resistin-induced ERK1/2 phosphorylation while simultaneously inhibiting hypertrophy [144].

Activation of endothelial nitric oxide synthase (eNOS) is a critical mechanism underlying apelin-induced vasodilation and blood pressure reduction [41]. This typically occurs downstream of PI3K/Akt activation, leading to eNOS phosphorylation, increased nitric oxide (NO) production, and subsequent activation of guanylate cyclase in smooth muscle cells. This cascade elevates cyclic GMP (cGMP) levels and causes relaxation [140].

PLC/PKC pathway—Phospholipase C (PLC) and protein kinase C (PKC), particularly PKCε, have been implicated in mediating the positive inotropic effects of apelin in cardiomyocytes [131].

Ion transport modulation—apelin signaling affects ion handling in cardiomyocytes. It can modulate Ca^2+^ transients and enhance myofilament Ca^2+^ sensitivity, potentially contributing to inotropy without necessarily increasing overall intracellular Ca^2+^ levels [140]. Mechanisms may involve the sarcolemmal Na^+^/H^+^ exchanger (NHE) and Na^+^/Ca^2+^ exchanger (NCX) [140]. In cerebral arteries, apelin inhibits large conductance Ca^2+^-activated K^+^ (BKCa) channels, counteracting NO-mediated relaxation [145].

Autophagy/Reticulophagy—apelin-13 has been shown to induce cardiomyocyte hypertrophy via activation of FAM134B-dependent reticulophagy (selective autophagy of the endoplasmic reticulum), potentially mediated by the Pannexin-1/P2X7 axis involving extracellular ATP signaling [146]. Autophagy regulation by apelin is also mentioned in other contexts [147].

β-Arrestin pathway and mechanotransduction—beyond classical G protein signaling, APLNR can recruit β-arrestins upon activation [131]. β-Arrestin recruitment typically leads to receptor desensitization and internalization, terminating G protein signaling; however, β-arrestins can also act as signaling scaffolds, initiating distinct, G protein-independent pathways [131]. Crucially, APLNR has been identified as a mechanosensor in cardiomyocytes [148]. Mechanical stretch, independent of ligand binding, activates APLNR signaling through a β-arrestin-dependent, G protein-independent pathway, which leads to pro-hypertrophic responses [148]. This dual signaling capability—ligand-mediated Gαi activation (often protective) versus stretch-mediated β-arrestin activation (pro-hypertrophic)—is a critical feature of APLNR function. Elabela also interacts with β-arrestin [131].

The multiplicity of ligands (apelin isoforms, ELA), the coupling to diverse intracellular pathways (Gαi, β-arrestin, PI3K/Akt, ERK, NO, PLC/PKC, ion channels, autophagy), and the unique ability to respond to both chemical and mechanical stimuli underscore the complexity of APLNR signaling. The ultimate physiological or pathological outcome of APLNR activation is highly dependent on the specific context, including the initiating stimulus (ligand type/isoform versus stretch), the cell type involved, and the prevailing physiological or pathological state. This inherent complexity likely contributes to some of the apparently contradictory findings reported in the literature, particularly regarding cardiac hypertrophy.

#### 3.1.1. Animal Studies

Hypertension-induced cardiac remodeling is a complex process involving multiple signaling pathways, among which the apelinergic system—comprising the apelin ligand and its receptor APLNR—plays a crucial role [62,149,150,151,152]. This system has been implicated in the pathophysiology of HHD through its regulatory effects on myocardial hypertrophy, fibrosis, vascular function, and neurohormonal interactions. Several studies have provided insights into the dynamic alterations in apelin/APLNR expression in hypertensive models, shedding light on its compensatory, regulatory, and therapeutic potential [62,151,152,153,154,155].

Iliev et al. reported an upregulation of APLNR expression in aged SHRs, particularly in the advanced stages of AH [62]. This suggests a compensatory mechanism in response to the depletion of its ligand, potentially highlighting the apelinergic system as a target for therapeutic intervention in HHD. Similarly, Najafipour et al. investigated two-kidney, one-clip (2K1C) hypertensive rats and observed dynamic changes in APLNR expression [151]. While myocardial APLNR mRNA and protein levels were reduced in the acute phase, they exhibited partial recovery during chronic AH. In contrast, aortic APLNR mRNA levels declined in both phases, with a significant reduction in protein expression only in the chronic phase, suggesting a complex regulatory mechanism in response to renovascular hypertension [151]. In 2K1C hypertensive rats, APLNR showed only a mild decrease in the chronic phase, possibly due to delayed receptor downregulation or translational inhibition. In contrast, both apelin and its receptor were markedly reduced in SHRs, likely due to the genetic, chronic nature of SHRs hypertension versus the acquired, secondary nature of 2K1C hypertension [151]. Additionally, AH leads to structural and functional alterations in the myocardium, including LVH and increased blood pressure [153]. Yeganeh-Hajahmadi et al. demonstrated that hypertensive male rats exhibited reduced apelin levels, alongside increased LVH and elevated blood pressure. Apelin administration led to a reduction in mean arterial pressure and left ventricular systolic pressure, an effect partially inhibited by opioid receptor antagonists [153]. This suggests an interaction between the apelinergic and opioid systems in regulating cardiovascular function. Moreover, hypertension-induced heterodimerization of APLNR and kappa-opioid receptor was normalized by apelin administration, further illustrating the intricate molecular interplay between these pathways [153]. Sekerci et al. further explored the tissue-specific effects of apelin/APLNR expression in hypertensive rats induced by nitric oxide synthase inhibition (L-NAME). They found increased apelin and APLNR expression in cardiac tissues but reduced levels in kidney tissues, indicating a differential, organ-specific adaptation of the apelinergic system in AH [152]. This finding underscores the need to consider tissue-specific responses when evaluating the therapeutic potential of apelin/APLNR modulation. Additionally, Zhang et al. reported increased apelin expression in the rostral ventrolateral medulla of SHRs, contributing to sympathetic overdrive and cardiovascular remodeling, suggesting a central role of apelin in AH pathogenesis [154].

**Figure 2:**
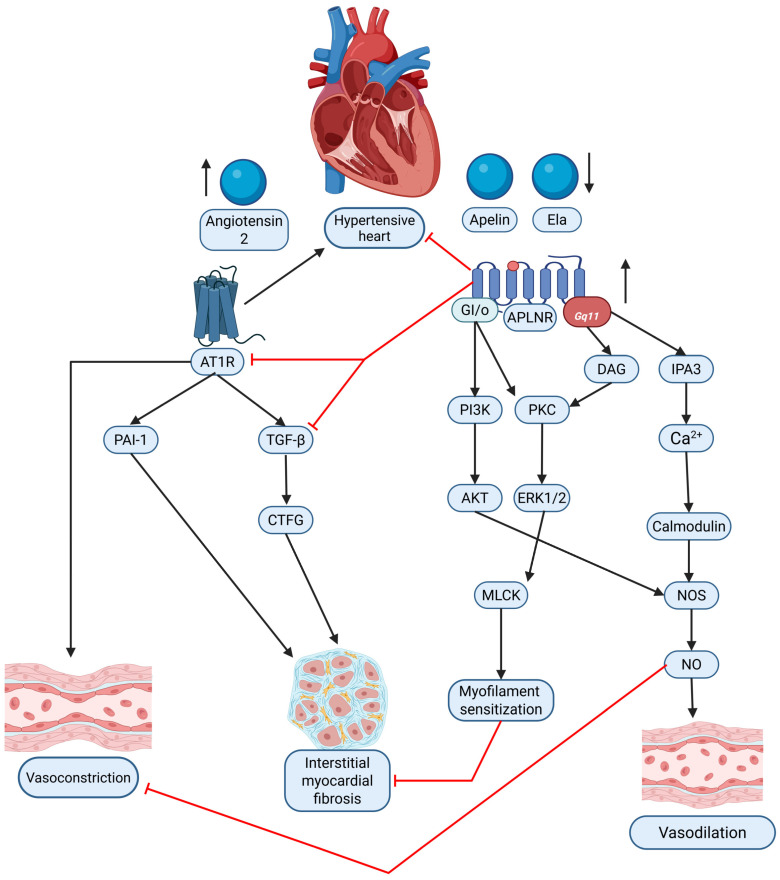
Graphical comparison of the interaction between the apelinergic system (apelin/Ela via APLNR) and the renin–angiotensin system (RAS, via Angiotensin II/AT1R) in hypertensive heart disease (HHD). The diagram illustrates the contrasting signaling pathways and their respective contributions to vasoconstriction, vasodilation, and interstitial myocardial fibrosis within the hypertensive heart. Black arrows—causation; red solid lines—inhibition. Original figure created with BioRender.com.

The RAAS plays a key role in hypertension-induced cardiac remodeling, and apelin has been identified as a counter-regulator of ANG II-mediated effects. Sato et al. demonstrated that apelin administration mitigated ANG II-induced cardiac fibrosis, hypertrophy, and dysfunction [155]. Apelin-knockout mice exhibited exacerbated cardiac damage under ANG II exposure, indicating the protective function of the apelinergic system. Notably, the loss of apelin disrupted the balance between angiotensin-converting enzyme (ACE) and ACE2, leading to ACE dominance and further amplifying ANG II’s deleterious cardiovascular effects [155]. This highlights apelin as a potential therapeutic agent for restoring cardiovascular homeostasis in hypertensive individuals. The interplay between the apelinergic system and the RAS in the context of HHD is summarized in Figure 2.

Based upon this review of the current literature, it is apparent that the apelin/APLNR system plays a multifaceted role in the hypertensive myocardium, functioning as both a compensatory mechanism and a potential therapeutic target. While AH induces alterations in APLNR expression across different tissues, the apelinergic system exerts cardioprotective effects by modulating myocardial hypertrophy, vascular function, and neurohormonal interactions [62,149,150,154,156]. Additionally, apelin’s ability to counteract ANG II-mediated damage underscores its significance in mitigating hypertension-induced cardiac remodeling [155]. Further research into targeted modulation of the apelinergic system may provide novel therapeutic avenues for HHD.

#### 3.1.2. Human Studies

Human studies further support the critical role of apelin/APLNR in HHD. Ye et al. identified a strong association between low serum apelin levels and LVH in essential hypertension (EH) patients, suggesting apelin’s role in mitigating pathological cardiac remodeling [157]. Moreover, Baysal et al. reported that untreated hypertensive patients had lower apelin levels than normotensive controls, while antihypertensive treatment increased apelin levels, indicating a regulatory role of the apelinergic system in blood pressure control [158]. Similarly, Hemmati et al. found that hypertensive patients had significantly lower apelin levels than healthy controls, with the highest levels in those receiving combination therapy, indicating its potential as a biomarker for AH management [159]. Gupta et al. examined plasma apelin-13 levels and genetic polymorphisms, showing significantly lower apelin levels in EH and acute coronary syndrome patients, particularly among females. Genetic analysis suggested a potential risk allele for ACS in women but no significant association with AH in males [160]. Ma et al. present a prospective observational study that investigated plasma Elabela levels in hypertensive patients—with and without heart failure (HF)—to assess its potential as a prognostic biomarker for major adverse cardiac events (MACE) [ma]. The study found that hypertensive patients had significantly lower plasma Elabela levels compared with healthy controls, and among hypertensive individuals, those with HF exhibited even lower levels, particularly in patients with impaired left ventricular systolic function [ma]. Furthermore, lower Elabela levels were associated with worse cardiac parameters, including reduced left ventricular ejection fraction, enlarged ventricular dimensions, and elevated pulmonary arterial pressure, as well as higher levels of established biomarkers such as BNP and troponin I, indicating more severe myocardial damage [161]. In survival analyses, patients with lower plasma Elabela levels experienced higher rates of HF readmission and MACE, and Cox regression analysis confirmed that decreased Elabela levels were an independent predictor of adverse outcomes. Since Elabela and apelin act on the same APLNR receptor and share cardioprotective effects—such as vasodilation, positive inotropy, and anti-fibrotic actions—this study suggests that a deficiency in apelinergic signaling may contribute to the progression of hypertensive heart disease, thereby positioning Elabela as a promising biomarker and potential therapeutic target for improving outcomes in this patient population [161].

Human studies confirm that reduced apelin levels are associated with left ventricular hypertrophy, blood pressure regulation, and cardiovascular risk, suggesting its potential as a biomarker and therapeutic target. However, in the current literature, there are significantly fewer studies concerning the role of the apelinergic system in the pathogenesis and progression of AH in humans. Further research into targeted modulation of the apelinergic system may provide novel therapeutic avenues for HHD. A thorough comparison of human and animal studies on the effects of the apelinergic system is presented in Table 8.

#### 3.1.3. Potential Therapeutic Implications

The apelin/APLNR system has been identified as a potential target for AH treatment due to its vasodilatory, cardioprotective, and anti-fibrotic properties [43,45,46,47,62,162]. Studies have shown that apelin and its analogs play a crucial role in regulating blood pressure, mitigating hypertensive myocardial damage, and promoting physiological cardiac remodeling [43,162,163,164].

Hypertension leads to cardiac hypertrophy, fibrosis, and oxidative stress, which contribute to myocardial dysfunction [162]. A significant body of evidence points to a functional antagonism and complex interplay between the apelinergic system and the RAAS, particularly the Ang II/AT1R axis. The two systems often exert opposing effects on cardiovascular function: apelin and Elabela generally promote vasodilation, lower blood pressure acutely, enhance cardiac contractility, and exhibit anti-fibrotic and anti-inflammatory properties [41,165], whereas Ang II acting via the AT1R typically causes vasoconstriction, elevates blood pressure, promotes pathological cardiac hypertrophy and fibrosis, and stimulates inflammation. Despite the structural homology and overlapping tissue distribution of APLNR and AT1R, they bind distinct ligands and mediate contrasting functional outcomes [41]. Experimental studies provide direct evidence that apelin signaling can counteract various detrimental actions of Ang II. For example, apelin infusion can blunt Ang II-induced vasoconstriction and pressor responses, preserving apelin-mediated vasodilation even during RAS activation such as sodium depletion or Ang II co-infusion [45,166]. Furthermore, apelin administration protects against Ang II-induced cardiovascular fibrosis in animal models by inhibiting the induction of pro-fibrotic factors like plasminogen activator inhibitor-1 (PAI-1) and potentially interfering with TGF-β1/Smad signaling [166]. On a cellular level, apelin can inhibit Ang II activation of pro-fibrotic signaling pathways in vascular smooth muscle cells and cardiac fibroblasts, such as Rho kinase activation and possibly PI3K/Akt signaling in fibroblasts [166]. In addition, apelin deficiency has been shown to exacerbate Ang II-induced cardiac dysfunction and adverse remodeling [147]. A critical point of interaction involves angiotensin-converting enzyme 2 (ACE2), which is a carboxypeptidase that plays a protective role in the RAS by degrading the vasoconstrictor Ang II into the vasodilator and anti-proliferative peptide Ang-(1–7) [167]. Intriguingly, ACE2 also acts as an enzyme that can cleave and potentially inactivate certain apelin isoforms (e.g., [Pyr1]apelin-13) [41]. Moreover, apelin/APLNR signaling has been shown to upregulate ACE2 expression and activity in the heart and potentially in other tissues, creating a complex feedback loop where apelin promotes ACE2, thereby enhancing the beneficial Ang-(1–7) arm of the RAS while simultaneously potentially increasing its own degradation [147]. In contrast, Elabela has been suggested to protect against pressure-overload heart failure possibly by suppressing ACE rather than ACE2 [147]. The net effect of manipulating apelin signaling, therefore, could depend significantly on the baseline activity of ACE2 and the overall state of the RAS. Pharmacological manipulation of the RAS also influences the apelin system. For instance, ACE inhibition with captopril in obese hypertensive rats led to increased adipose tissue expression of both the beneficial Ang-(1–7)/Mas receptor axis and the apelin/APLNR system, along with blood pressure reduction. Similarly, angiotensin receptor blockers such as losartan may exert some of their beneficial anti-fibrotic effects partly through pathways involving apelin/APLNR signaling, including Akt/eNOS activation [142]. Pyr1-apelin-13 has been shown to reduce ANG II-induced cardiac hypertrophy, fibrosis, and oxidative stress, highlighting its therapeutic potential [162]. While acute studies often show apelin counteracting Ang II, the chronic interaction appears more complex; for example, chronic co-administration of apelin-13 failed to prevent Ang II-induced hypertension, cardiac hypertrophy, or fibrosis in rats, a discrepancy that might be explained by the short half-life of apelin-13, receptor desensitization upon continuous stimulation, or the activation of counter-regulatory mechanisms over time [162]. These findings suggest that apelin counteracts the harmful effects of the RAAS, a key driver of AH and cardiac remodeling. Future research in this area could provide deeper insights into the complex interplay between the apelinergic system and the RAAS. A more comprehensive understanding of the synergy between these two systems may pave the way for optimized combination therapies, integrating ACE inhibitors or ANG II blockers with apelinergic system modulators for enhanced antihypertensive treatment. Apelin has also been demonstrated to lower blood pressure and restore plasma apelin levels. Apelin administration significantly reduced blood pressure and upregulated apelin/APLNR gene expression, further supporting its role in modulating the RAAS and serving as a potential therapeutic approach for AH [43]. Additionally, apelin-13 inhibits hypertrophic signaling and inflammation via the Hippo pathway, reducing pathological cardiac changes and preserving myocardial function [163]. Furthermore, the vasodilatory effects of apelin and its analogs contribute to improved cardiac function without adverse effects, making them promising candidates for antihypertensive therapy [164]. These effects help reduce systemic vascular resistance and alleviate the excessive cardiac workload caused by AH. 

The multifaceted roles of the apelin/APLNR system in cardiovascular regulation and its dysregulation in hypertension and heart failure make it an attractive therapeutic target for HHD. In HHD, reduced endogenous apelin/Elabela levels and impaired APLNR receptor function contribute to the development of pathological processes; so, restoring physiological balance by augmenting APLNR signaling is a logical strategy to regain cardiovascular homeostasis [168]. Enhancing APLNR signaling may offer multifaceted benefits, including blood pressure reduction via nitric-oxide-dependent vasodilation [41], improved cardiac contractility—potentially benefiting patients with heart failure with reduced ejection fraction while avoiding the adverse effects of conventional inotropes [36]—and anti-fibrotic effects that could reverse myocardial fibrosis [41]. Moreover, increasing APLNR signaling can provide direct protection to cardiomyocytes by inhibiting apoptosis and promoting cell survival under stress conditions [141], and it may counteract the detrimental actions of angiotensin II, thereby opposing the overactive renin–angiotensin system [147].

Several pharmacological strategies have been explored to modulate the apelin/APLNR system. Direct administration of native peptides such as [Pyr1]apelin-13 or Elabela has shown beneficial acute effects—improving cardiac contractility, inducing vasodilation, and lowering peripheral resistance—but their clinical application for chronic conditions is limited by an extremely short plasma half-life and rapid enzymatic degradation [41,164]. As a result, modified peptide analogues, which exhibit improved stability, and non-peptidic small molecule agonists that are orally bioavailable have been developed [41]. An especially promising approach involves the creation of biased agonists that preferentially activate the protective Gαi signaling pathway while minimizing β-arrestin recruitment, which is associated with receptor desensitization and pathological hypertrophy due to mechanical stretch [41,169]. For instance, peptide analogues like MM07 and small-molecule candidates such as CMF-019 have shown enhanced vasodilatory and inotropic benefits in preliminary studies, with early-phase clinical trials of compounds like BMS-986224 demonstrating initial safety and pharmacokinetic profiles [41,170].

Exercise has been identified as a physiological modulator of the apelin/APLNR system [171,172]. Exercise training leads to an increase in IGF-1 release, activating the AMPK and AKT signaling pathways, which upregulate APLNR and apelin mRNA expression. These molecular changes shift myocardial remodeling from a pathological to a physiological state in SHRs. Blocking IGF1R and APLNR inhibited these cardioprotective effects, demonstrating the importance of the apelinergic system in reducing hypertension-induced cardiac damage [171]. Further studies confirmed that exercise training reduces systolic blood pressure and upregulates apelin/APLNR expression, reinforcing the beneficial role of apelin in hypertensive myocardium [172].

Despite these promising strategies, several challenges remain. The short half-life and rapid degradation of native peptides necessitate the development of more stable analogues or small-molecule agonists suitable for chronic administration [41]. Moreover, fully understanding the differential downstream effects of G protein versus β-arrestin signaling is crucial to safely harnessing the protective aspects of APLNR activation, as the receptor also functions as a mechanosensor under chronic pressure overload conditions [41]. Future research should focus on robust long-term preclinical models, detailed studies of signaling mechanisms, and well-designed clinical trials to validate the efficacy and safety of these novel agents in HHD, as well as on developing reliable biomarkers for patient stratification. The therapeutic implications of the apelinergic system are summarized in Table 9.

Overall, the apelin/APLNR system plays a crucial role in AH treatment. Apelin and its analogs show potential in lowering blood pressure, preventing hypertensive myocardial remodeling, and enhancing cardiovascular adaptation through exercise-induced upregulation. Given its cardioprotective, vasodilatory, and anti-inflammatory properties, the apelinergic system represents a promising therapeutic target. Future research should focus on optimizing apelin analogs and evaluating their long-term efficacy in hypertensive patients.

### 3.2. Role of VEGF/VEGFR Pathway in the Context of AH and Hypertensive Myocardium

The VEGF family of signaling proteins and their receptors plays a pivotal role in forming, maintaining, and modulating blood and lymphatic vessels, which is essential for vascular function. The VEGF ligands belong to the PDGF superfamily and are structurally characterized by a cystine-knot motif formed by conserved cysteine residues; they usually function as secreted, disulfide-linked homodimers, although heterodimers may also form [173]. The main mammalian members include VEGF-A, VEGF-B, VEGF-C, VEGF-D, and Placenta Growth Factor (PlGF) [174]. VEGF-A, the most extensively studied, is critical for vasculogenesis, angiogenesis, and vascular permeability. It exists in multiple isoforms (e.g., VEGF-A121, VEGF-A165, VEGF-A189, VEGF-A206) generated through alternative splicing, with each isoform exhibiting distinct biochemical properties based on their affinity for heparin and heparan sulfate proteoglycans, which, in turn, affects their diffusibility and localization [54,175]. In addition, alternative splicing at exon 8 produces the VEGFxxxb family, which generally exhibits reduced receptor activation and may modulate the pro-angiogenic actions of the VEGFxxxa isoforms [176]. VEGF-B, which primarily binds to VEGFR-1 and Neuropilin-1, is abundant in metabolically active tissues such as the heart and skeletal muscle, where it regulates endothelial cell survival and fatty acid metabolism [174,177]. VEGF-C and VEGF-D are the principal regulators of lymphangiogenesis via VEGFR-3 but, after proteolytic processing, can also activate VEGFR-2 to contribute to angiogenesis, inflammation modulation, and fibrogenesis [176,178]. PlGF, which binds primarily to VEGFR-1, may form heterodimers with VEGF-A and is implicated in pathological angiogenesis and inflammation [174,176,179].

VEGF ligands exert their effects by binding to transmembrane receptor tyrosine kinases (RTKs) on various cells, primarily on endothelial cells, including VEGFR-1, VEGFR-2, and VEGFR-3 [175,178]. VEGFR-1, despite its high affinity for VEGF-A, PlGF, and VEGF-B, has weak intrinsic tyrosine kinase activity and often acts as a decoy receptor that modulates the availability of VEGF-A for the more potent VEGFR-2 while also participating in vascular growth and inflammation when activated by specific ligands [174,176]. VEGFR-2 is considered the principal mediator of the biological effects of VEGF-A, driving endothelial proliferation, migration, survival, angiogenesis, and vascular permeability [175,178]. VEGFR-3, mainly expressed in lymphatic endothelial cells, is essential for lymphangiogenesis but can also interact with VEGFR-2 during active angiogenesis [55,178]. Co-receptors such as neuropilins (NRP-1 and NRP-2) and heparan sulfate proteoglycans (HSPGs) further modulate signaling by facilitating ligand binding and receptor activation [174,178,179].

Activation of VEGFRs occurs through ligand-induced dimerization and autophosphorylation of specific tyrosine residues, which serve as docking sites for adaptor proteins and initiate multiple intracellular signaling pathways [180,181]. One key pathway is the PLCγ pathway, where PLCγ hydrolyzes PIP2 into IP3 and DAG—IP3 increases intracellular Ca^2+^ and DAG activates PKC, leading to the activation of the MAPK (ERK1/2) cascade that promotes endothelial cell proliferation and migration [181]. Another major pathway is the PI3K/Akt pathway; activation of VEGFR-2 recruits PI3K, which produces PIP3 and subsequently activates Akt. Akt phosphorylates eNOS, increasing nitric oxide (NO) production that is critical for vasodilation and vascular permeability [177,182,183]. Additionally, Src family kinases are activated and contribute to changes in cell adhesion and migration, while other adaptors, including TSAd, SHB, NCK, FAK, paxillin, and p38 MAPK, further diversify the cellular responses to VEGF signaling [182]. The specific outcome of VEGF/VEGFR activation is highly context-dependent, relying on the particular isoform involved, the type of receptor dimer (homodimer or heterodimer), the engagement of co-receptors, and the cellular environment, all of which are key to understanding its role in both normal vascular physiology and diseases such as HHD [55,176].

#### 3.2.1. Animal Studies

The VEGF/VEGFR pathway plays a central role in the hypertensive myocardium, mediating angiogenesis, cardiac remodeling, and the transition from adaptive hypertrophy to HF [62,63,149,150,184,185]. Pressure overload and AH induce VEGF expression during the compensatory phases of hypertrophy, with varying effects depending on age, disease stage, and VEGF receptor interactions [62,149,150,186]. Studies using various animal models of hypertension and pressure overload (e.g., transverse aortic constriction, abdominal aortic banding, angiotensin II infusion, spontaneously hypertensive rats) have investigated changes in cardiac VEGF/VEGFR signaling, yielding somewhat varied but informative results. Several studies report an upregulation of VEGF-A mRNA and/or protein expression in the myocardium during the development of pressure-overload-induced hypertrophy [181,182]. For instance, transverse aortic constriction (TAC) in mice led to a nearly three-fold increase in VEGF-A transcript levels [178]. Similarly, infrarenal aortic banding in rats caused an increase in both VEGF mRNA and protein [187]. This upregulation is often considered a compensatory response—potentially driven by mechanical stretch, neurohormonal factors such as Ang II, or relative hypoxia in the hypertrophying tissue, which aims to promote angiogenesis to match the increased myocardial mass [182]. However, conflicting findings exist. One study using TAC in mice with β-adrenergic receptor deletions found no increase in VEGF expression during compensated hypertrophy, although sham surgery itself increased VEGF compared to non-operated controls, highlighting potential confounding factors [188]. Another study reported decreased VEGF mRNA in cardiomyopathic hamsters treated with valsartan, an ARB [189]. The timing of measurement and the specific model used (compensated versus decompensated hypertrophy) likely influence these results [178]. Jesmin et al. demonstrated that VEGF expression fluctuates with age in hypertensive rat models, showing an initial increase in young SHRs followed by a decline in older animals, while SHRs prone to stroke (SHRSP) maintained elevated levels into older age, particularly in the LV [184]. Duan et al. observed that upregulation of VEGF-A increases the capillary-to-cardiomyocyte ratio but results in a net reduction in CD due to the disproportionate growth of cardiac myocytes, contributing to progressive interstitial fibrosis and reduced myocardial contractility, thereby expediting the shift to HF [185]. Gu et al. found a five-fold increase in VEGF-A expression in the LV of 18-week-old (4 months and a half) SHRs compared to normotensive controls [63]. Mcanish et al. investigated VEGF188 mRNA expression in relation to cardiac hypertrophy and coronary flow in SHRs compared to controls [190]. While SHRs exhibited elevated blood pressure, increased heart weight-to-body weight ratios, and impaired coronary flow at 12 weeks (4 months), the maximal coronary flow normalized by 28–32 weeks (7–8 months) despite persistent hypertrophy. The VEGF188 mRNA levels were initially similar between groups but increased three-fold in SHRs by 32 weeks (8 months), suggesting that VEGF188 upregulation may compensate to preserve coronary flow during hypertrophy progression [190]. Stanchev et al. reported an initial elevation in VEGF-A levels in both ventricles of 6-month-old SHRs, followed by a significant depletion in VEGF-A expression, particularly in the LV, by 12 months [70]. This progression correlates positively with CD as AH advances, highlighting the crucial role of VEGF-A in maintaining compensatory mechanisms during the early stages of AH [70]. The subsequent depletion of VEGF-A is associated with the transition from AH to HF, underscoring its pivotal role in preserving myocardial function during hypertensive remodeling. [70]. In a recent study, Iliev et al. assessed the topological distribution of VEGF-A in the heart walls of 6- and 12-month-old SHRs [62]. They confirmed the results of Stanchev et al. and provided further details of the regional changes in VEGF-A expression within the endocardium, myocardium, and epicardium, with the most pronounced depletion observed in the myocardium of the LV in 12-month-old SHRs [62]. In contrast, the RV showed no significant changes in VEGF-A levels across all three layers [62]. Conversely, in normotensive controls, Iliev et al. noted a significant elevation in VEGF-A in all three layers of the heart wall in both ventricles with age progression [62]. Moreover, VEGF-A plays a critical role in the compensatory mechanisms during the progression of AH [62,70,149,191]. Reduced cardiac angiogenesis is a significant factor in the transition from adaptive cardiac hypertrophy to HF [62,70,149,192,193]. The importance of VEGF in sustaining cardiac function under pressure overload is underscored by findings that its inhibition accelerates this transition [62,70,149,194].

The regulatory mechanisms controlling VEGF expression under hypertrophic stress are multifaceted. Leychenko et al. found that cyclic mechanical stretch in cardiomyocytes induces VEGF secretion through the NFκB pathway, emphasizing VEGF’s importance in responding to mechanical stress [186]. In contrast, Duan et al. identified XBP1, a transcription factor involved in the unfolded protein response, as critical for VEGF-A regulation [185]. Silencing XBP1 reduced VEGF levels, impairing angiogenesis and accelerating cardiac dysfunction, thereby highlighting the interplay between cellular stress responses and VEGF signaling [185]. Zaw et al. demonstrated that secretin knockout results in AH and cardiac hypertrophy, characterized by decreased plasma VEGF levels but increased cardiac tissue VEGF levels. This finding suggests a compensatory mechanism triggered in response to cardiac remodeling [195]. The study also noted reductions in NO and increased aldosterone levels, which may contribute to the observed AH and cardiac changes. Additionally, gene expression analysis showed upregulation of ANGII receptors and myosin heavy chain genes, with a decrease in eNOS expression, highlighting the complex interplay between VEGF signaling, AH, and cardiac remodeling [195].

The roles of VEGF-A and its receptors VEGFR-1 and VEGFR-2 in cardiac remodeling are particularly critical. VEGFR-2 is central to VEGF-mediated angiogenesis, while VEGFR-1 regulates VEGF availability and bioactivity. Changes in receptor expression are also observed in hypertrophic hearts subjected to pressure overload. VEGFR-1 expression, at both the mRNA and protein levels, has been reported to increase in these models [196]. Additionally, the soluble form, sVEGFR-1, may be upregulated, potentially sequestering VEGF-A and limiting its bioavailability for VEGFR-2 signaling [197]. Data on VEGFR-2 expression, however, are more variable; while some studies report upregulation [198], others show no change or even downregulation, particularly in later stages or under specific conditions [196]. Importantly, changes in receptor expression do not always reflect changes in receptor activity. For example, studies examining VEGFR-2 phosphorylation—a marker of receptor activation—have found that phosphorylation can be increased in response to certain stimuli or interventions in hypertrophied hearts [182], but may be impaired under other circumstances [196]. In one instance, ablation of VEGFR-1 signaling led to altered phosphorylation of downstream targets such as mTOR and PKCα during pressure overload, suggesting the existence of complex cross-talk between these receptors [199]. Moreover, endothelial-specific deletion of PTP1B, which is a negative regulator of VEGFR-2, enhanced VEGFR-2 phosphorylation and conferred protection against pressure-overload-induced failure [197]. Kaza et al. showed that in hypertrophied hearts, VEGFR-1 and its soluble isoform sVEGFR-1 were upregulated, sequestering VEGF and limiting its availability for VEGFR-2-mediated angiogenesis [196]. Treatment with PlGF released VEGF-A from sVEGFR-1, enhancing VEGFR-2 activation, promoting angiogenesis, and improving myocardial function. This suggests that targeting sVEGFR-1 may mitigate maladaptive remodeling and preserve cardiac function in hypertrophy [196]. Similarly, Mei et al. demonstrated that VEGFR-1 deficiency exacerbates cardiac hypertrophy and dysfunction by disrupting VEGF-mediated angiogenesis, further underscoring VEGFR-1’s protective role in pressure overload [200]. Adapala reveals that endothelial TRPV4 suppression enhances VEGF-A and VEGFR2-mediated angiogenesis in the myocardium. TRPV4 knockout increased VEGFR2 expression and activation, promoting coronary angiogenesis and CD [201]. This response was mediated through YAP signaling and the Rho/Rho kinase/LATS1/2 pathway, suggesting that TRPV4 normally inhibits VEGF-A/VEGFR2 signaling, and its deletion enhances proangiogenic responses, benefiting cardiac function under hypertrophic stress [201].

Beyond VEGF-A, other VEGF family members contribute to hypertensive remodeling. VEGF-B promotes metabolic adaptation and vascular remodeling but may also exacerbate maladaptive processes under stress. Samuelsson et al. showed that VEGF-B overexpression leads to left ventricular hypertrophy, metabolic exhaustion, and fibrosis, suggesting a dual role in adaptation and pathology [202]. Karpanen et al. reported that VEGF-B167 interacts with VEGFR-1 and integrates into myocardial blood vessels and cardiomyocytes, playing a role in cardiac hypertrophy and vascular remodeling. VEGF-B167 reduced vessel density, potentially due to decreased VEGFR-2 expression, impairing angiogenic signaling. These findings suggest VEGF-B167 and its interactions with VEGFRs influence hypertrophy, vascular remodeling, and energy metabolism [203]. VEGF-C and VEGFR-3 are involved in lymphangiogenesis and the regulation of cardiac edema. Yang et al. highlight that high salt intake in SHRs increases VEGF-C expression and VEGFR-3 activation, promoting lymphangiogenesis in the myocardium [44]. This upregulation of VEGF-C/VEGFR-3 signaling was linked to enhanced cardiac hypertrophy, fibrosis, and macrophage infiltration, exacerbating left ventricular remodeling. These findings suggest that VEGF-C-mediated lymphangiogenesis contributes significantly to hypertension-induced cardiac remodeling, making it a potential therapeutic target for hypertension-related cardiac diseases [44]. Furthermore, Bai et al. demonstrate that VEGF-C and VEGFR-3 play a significant role in cardiac remodeling and lymphangiogenesis induced by ANG II infusion [204]. ANG II upregulated VEGF-C and VEGFR-3, promoting cardiac lymphangiogenesis and increasing lymphatic vessel permeability, contributing to cardiac edema. VEGFR-3 knockdown worsened ANG II-induced cardiac edema, hypertrophy, and dysfunction by impairing lymphangiogenesis and amplifying fibrosis, oxidative stress, and inflammation [204]. These findings highlight VEGFR-3′s critical role in regulating lymphangiogenesis and protecting against cardiac remodeling and edema induced by ANG II [204].

Collectively, these findings underscore the complex, multifaceted roles of the VEGF/VEGFR pathway in the hypertensive myocardium. Dysregulation of this pathway accelerates the progression from compensatory hypertrophy to HF, with VEGF bioavailability, receptor activation, and downstream signaling pathways playing critical roles. Interventions targeting this pathway, such as enhancing VEGF activity through PlGF or modulating VEGFR signaling, hold promise for mitigating pathological remodeling and improving outcomes in HHD.

#### 3.2.2. Human Studies

In contrast to the extensive data from animal studies, the current literature includes relatively few studies examining the role of the VEGF/VEGFR pathway in humans with AH. Duan et al. demonstrated that elevated XBP1 levels in human hypertrophic and failing hearts were associated with increased VEGF-A expression, suggesting that XBP1 regulates VEGF-A to drive myocardial angiogenesis and support the progression of hypertrophy [185]. This indicates that VEGF-A acts as an adaptive mechanism to promote vascular growth and protect the myocardium during hypertrophic stress, underlining its essential role in maintaining myocardial function [185]. Weiss et al. demonstrated the potential therapeutic role of VEGF-1 induction in cardiac myocytes [205]. They found that Prostaglandin E1 (PGE1) significantly increased VEGF-1 production, particularly the VEGF165 splice variant, in human adult cardiac myocytes via a cAMP-dependent mechanism. Importantly, this VEGF-1 was biologically active, as it promoted endothelial cell proliferation and tube formation—key processes in angiogenesis [205]. These findings underscore the potential of VEGF-1 to enhance myocardial angiogenesis and improve blood flow, suggesting that PGE1-induced VEGF-1 expression may serve as a valuable therapeutic strategy for ischemic myocardium [205]. Circulating VEGF levels: Some studies in hypertensive patients have reported elevated plasma VEGF levels compared to normotensive controls, and these elevated levels correlate with cardiovascular risk scores while being reduced by intensive risk factor management [206]. This may reflect endothelial damage or activation, or it could be part of a systemic compensatory response.

**Figure 3:**
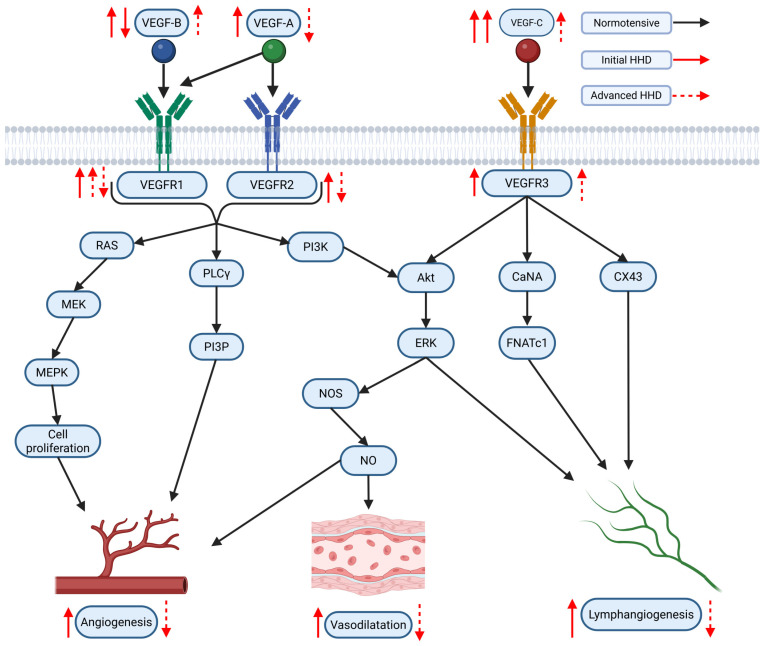
Graphical representation of the VEGF/VEGFR signaling network in the context of hypertensive heart disease (HHD), highlighting reported alterations in expression/activity associated with initial and advanced stages of HHD. Original figure created with BioRender.com.

Figure 3 maps the VEGF/VEGFR signaling network within the setting of HHD, outlining changes in expression and activity linked to disease development. It distinguishes between molecular alterations occurring at early versus advanced stages, offering insight into the pathway’s evolving role with the progression of the disease.

VEGF/VEGFR in failing human hearts: In analyses of myocardial tissue from explanted failing hearts due to dilated cardiomyopathy (DCM) or ischemic cardiomyopathy (ICM), altered VEGF signaling has been observed. In DCM, VEGF-A mRNA and protein, as well as VEGFR-1, are often downregulated and correlate with reduced capillary density [192]. In contrast, ICM hearts sometimes show upregulated VEGF isoforms, such as VEGF-A and VEGF-C, although receptor signaling may be impaired [192]. In diabetic patients with chronic coronary heart disease, myocardial biopsies have revealed significantly higher levels of VEGF mRNA and protein compared to non-diabetic CHD patients; however, these patients often exhibit paradoxically lower expression and phosphorylation (activation) of VEGFR-2 (Flk-1), suggesting receptor downregulation or desensitization despite high ligand levels [207]. These findings indicate that the underlying etiology of heart failure and comorbidities like diabetes significantly impact cardiac regulation of the VEGF pathway.

VEGF inhibitor-induced hypertension: A crucial line of evidence comes from oncology, where drugs targeting the VEGF pathway—such as VEGF inhibitors like bevacizumab or VEGFR inhibitors like sunitinib and pazopanib—are widely used. A major and frequent side effect of these therapies is the development or worsening of hypertension, which occurs in a substantial proportion (20–90%) of patients, often rapidly after treatment initiation [191]. This “on-target” toxicity underscores the critical role of constitutive VEGF signaling in maintaining normal blood pressure and vascular homeostasis in humans. The underlying mechanisms include reduced nitric oxide production, increased endothelin-1 levels, oxidative stress, and microvascular rarefaction [183]. Studies using these inhibitors have directly demonstrated reduced microvascular density in humans [208].

Conversely, Felmeden et al. highlighted the pathological elevation in VEGF levels in hypertensive patients and its association with cardiovascular risk [206]. They found that plasma VEGF levels were higher in hypertensive individuals, especially those with higher cardiovascular risk profiles [206]. VEGF levels positively correlated with age, blood pressure, and cardiovascular risk scores, indicating its role in endothelial dysfunction and hypertension-related cardiovascular complications [206]. Moreover, intensive cardiovascular risk management effectively reduced VEGF levels, suggesting that VEGF could be both a biomarker and a therapeutic target for hypertensive cardiovascular disease [206]. Additionally, the study noted a relationship between VEGF, von Willebrand factor (vWf), and soluble Flt-1 (sFlt-1), further linking VEGF to endothelial damage and dysfunction in AH [206].

In contrast to these findings, Vyzantiadis observed lower VEGF levels in hypertensive patients compared to healthy controls, highlighting a potentially impaired VEGF-mediated response in AH [209]. The VEGF levels in healthy individuals positively correlated with NO levels, which are critical for endothelial function and vascular health [209]. However, this VEGF-NO relationship was absent in untreated hypertensive patients, suggesting that AH disrupts the interaction between VEGF and NO, potentially contributing to endothelial dysfunction and vascular remodeling [209].

Shimizu provided a genetic perspective on VEGF activity in hypertension-related cardiovascular conditions [210]. The study focused on the VEGF polymorphism rs3025039, which was associated with reduced VEGF activity [210]. Intriguingly, this polymorphism was inversely correlated with atherosclerosis in hypertensive older Japanese individuals, indicating that lower VEGF-mediated angiogenesis might confer protection against atherosclerosis [210]. These findings contrast with the conventional view that AH exacerbates atherosclerosis and suggest that diminished angiogenesis, potentially through reduced VEGF activity, might mitigate atherosclerotic progression in certain contexts [210].

The role of VEGF in the human hypertensive myocardium is multifaceted, balancing adaptive and maladaptive responses. On one hand, VEGF promotes myocardial angiogenesis, mitigating ischemic damage and supporting tissue adaptation under hypertrophic stress [185,205]. On the other hand, dysregulated VEGF signaling, as seen in hypertensive patients, may exacerbate endothelial dysfunction, as evidenced by its correlations with vWf and sFlt-1 [206] and the impaired VEGF-NO interaction in untreated AH [209]. Additionally, genetic variations in VEGF activity, such as the rs3025039 polymorphism, highlight its nuanced role in atherosclerosis risk modulation [210]. Collectively, these studies suggest that therapeutic strategies targeting VEGF signaling need to account for its context-dependent effects, aiming to restore balanced angiogenesis while minimizing endothelial dysfunction in hypertensive myocardium. Further research is necessary to clarify the interplay between VEGF, its receptors, and downstream pathways in AH-related cardiac remodeling.

The disparity between the number of human and animal studies exploring the role of VEGF/VEGFR signaling in the hypertensive myocardium underscores the need for further research to elucidate the pathway’s function in the pathophysiology of AH and its progression to HF in humans. Expanding human-focused research on VEGF/VEGFR dynamics could yield valuable insights into therapeutic strategies targeting this pathway, potentially paving the way for more effective treatments to mitigate the progression of hypertensive cardiovascular disease and improve patient outcomes. An in-depth comparison of human and animal studies on VEGF/VEGFR signaling is presented in Table 10.

#### 3.2.3. Potential Therapeutic Implications

The VEGF/VEGFR pathway has significant therapeutic implications in managing hypertensive myocardium, playing a dual role in promoting adaptive remodeling and contributing to adverse cardiovascular events when inhibited [61,211,212,213,214]. Therapeutic interventions targeting this pathway provide opportunities to improve outcomes in pressure-overload-induced cardiac remodeling and hypertensive cardiovascular conditions. Shu et al. demonstrated the therapeutic potential of trimetazidine (TMZ), which promotes angiogenesis and enhances cardiac function in pressure-overload-induced cardiac hypertrophy. By upregulating VEGF-A expression through the Akt-HSF1-VEGF signaling pathway, TMZ increased VEGF and CD31 levels, highlighting its role in facilitating angiogenesis and potentially mitigating hypertensive remodeling [215]. Similarly, Belcik et al. emphasized VEGF’s critical role in cardiovascular homeostasis. Their study showed that inhibiting VEGF with G6-31 induced LVH and functional impairments, including increased wall thickness and hypertrophic markers, while co-administration of ramipril alleviated these changes, underscoring VEGF’s protective function in stress adaptation [216].

Exercise has emerged as a potent modulator of VEGF-mediated processes which might also leverage the VEGF pathway for therapeutic benefit. Tian et al. demonstrated that exercise restores VEGF-A and HIF-1α levels, enhancing angiogenesis and improving cardiac function in hypertrophic myocardium. These findings emphasize the therapeutic potential of exercise-induced VEGF modulation in preventing pathological remodeling [217]. Husain et al. found that exercise training enhanced VEGF expression and NO bioavailability, mitigating oxidative stress and endothelial dysfunction caused by chronic AH. This normalization of VEGF signaling underlines the value of physical activity in countering hypertensive cardiovascular damage [218]. Similarly, Yazawa et al. reported that perindopril restored VEGF levels and CD in chronic heart failure (CHF) models, reversing adverse remodeling and supporting myocardial angiogenesis [67]. Belabbas et al. further confirmed the benefits of exercise, showing that physical activity normalized vessel density and enhanced angiogenesis in ANG II-induced AH, even under hypertensive stress [219].

VEGF inhibitors (VEGFIs) are strongly associated with the development of AH, a well-documented adverse effect observed across various studies. Blood pressure increases following VEGFI therapy are often significant, frequently exceeding 150/100 mmHg, and the severity of AH is dose-dependent, with greater risk when multiple VEGFIs are combined [211,212,213]. Bevacizumab, a widely used VEGFI, has been associated with a 20–30% higher incidence of AH than expected [220]. The importance of monitoring blood pressure in these patients is underscored by findings that home blood pressure monitoring detects more cases of AH than clinical measurements [220]. Ambulatory blood pressure monitoring (ABPM) has demonstrated a rapid onset of AH following VEGFI therapy, with over 90% of patients exhibiting increased blood pressure. Additionally, ABPM revealed disrupted circadian blood pressure patterns, further elevating the risk of cardiovascular events [61,214]. For example, in sunitinib-treated renal cell carcinoma patients, blood pressure increases were observed within the first two weeks of therapy and persisted until treatment cessation [61].

Additionally, VEGFIs are strongly associated with AH, with a rapid onset often observed shortly after initiating therapy [221]. The severity of VEGFI-induced AH is dose-dependent, and its occurrence is influenced by factors such as pre-existing hypertension and VEGFI type [214,221,222]. Mechanistically, VEGFIs disrupt vascular homeostasis by impairing NO signaling, leading to decreased vasodilation and increased vasoconstriction. These vascular changes, along with alterations in endothelial function, are key drivers of elevated blood pressure.

AH induced by VEGFIs appears independent of proteinuria, as renal injury is more closely linked to VEGFI dose than to the hypertensive response [214]. This suggests that while VEGFIs impact vascular tone, renal dysfunction does not directly drive the observed blood pressure elevations. VEGF receptor inhibitors (VEGFRis) are particularly potent, causing faster and more pronounced AH compared to VEGFIs. Key pathways implicated in VEGFI-induced hypertension include signaling and NO/eNOS dysregulation, both of which contribute to the hemodynamic effects observed during treatment [214,221,222].

The rapid onset of VEGFI-induced AH and its resolution upon discontinuation highlight vascular tone changes as a central mechanism. These include decreased vasodilation and increased vasoconstriction, which are direct effects of VEGFI therapy. Notably, hypertension induced by VEGFI treatment is increasingly recognized as a biomarker of therapeutic efficacy as cancer patients who develop AH during therapy often achieve better outcomes [223].

Further insights into VEGFI and VEGFR inhibitor (VEGFRi)-induced AH were provided by Kuang et al., who demonstrated that VEGFRis have a stronger tendency to induce both systolic and diastolic hypertension compared to VEGFIs, with higher reported odds ratios (RORs) and faster onset times [222]. VEGFRis generally produced more severe and variable blood pressure increases, as confirmed in animal models where systolic, mean, and diastolic blood pressures were significantly elevated [222]. VEGFRis were also strongly associated with hypertension-related adverse events across cancer types, particularly in head and neck squamous cell carcinoma [222]. Mechanistically, the MAPK signaling pathway and impaired NO regulation via eNOS were identified as key contributors to VEGFI- and VEGFRi-induced AH [222].

Overall, the VEGF/VEGFR pathway serves as a critical target in managing hypertensive myocardium. While therapies like TMZ, perindopril, and exercise enhance VEGF signaling to support cardiac function and angiogenesis, VEGFI treatments require careful monitoring due to their hypertensive effects. Future research should focus on balancing VEGF pathway modulation to optimize therapeutic outcomes while minimizing adverse cardiovascular events. Moreover, future research should explore the potential benefits of combining VEGF signaling promotion with existing antihypertensive therapies to achieve improved outcomes.

### 3.3. Role of NO/NOS Signaling in the Context of AH and Hypertensive Myocardium

The NO/NOS signaling pathway plays a crucial role in cardiovascular health, and its dysregulation in hypertension and HHD is a key contributor to disease pathophysiology. eNOS dysfunction—through reduced activity and uncoupling—leads to impaired vasodilation, increased oxidative stress, and adverse cardiac remodeling [224,225]. In addition, nNOS, found in cardiac myocytes and autonomic centers, influences heart contractility and sympathetic tone. While nNOS may compensate for eNOS dysfunction by maintaining NO production and exerting anti-hypertrophic effects [226,227,228], it too can become uncoupled under oxidative stress, contributing to ROS generation and pathology [229,230,231]. Under normal conditions, iNOS expression in the heart is minimal, but it becomes markedly upregulated by inflammatory cytokines during severe cardiac stress. The resulting high levels of NO from iNOS can lead to nitrosative stress, mitochondrial dysfunction, and adverse remodeling [232].

In HHD, dysregulated NO/NOS signaling interacts with several key pathophysiological pathways, including the RAAS, oxidative stress/mitochondrial dysfunction, and inflammation/fibrosis. RAAS activation, particularly through Ang II, impairs NO signaling by stimulating NADPH oxidases to increase ROS production, which both scavenges NO and oxidizes BH4, leading to eNOS uncoupling [233,234]. Ang II may also suppress eNOS expression or activity directly [235]. Although NO may inhibit renin release and support AT2 receptor-mediated protective effects the overall impact of RAAS activation diminishes NO bioavailability [228].

Oxidative stress, resulting from an imbalance between ROS production and antioxidant defenses, further disrupts NO signaling. Key ROS sources include NADPH oxidases, xanthine oxidase, and uncoupled NOS isoforms [236], while mitochondria serve as both a source and target of ROS [233]. Excess ROS react with NO to form peroxynitrite, a potent oxidant that damages cellular components and further oxidizes BH4, thus perpetuating eNOS uncoupling and reducing NO levels [234,237]. Additionally, mitochondrial damage—marked by structural alterations, ETC inefficiency, and mtROS leakage—creates a feedback loop of cellular dysfunction and energy deficits [238]. Reduced NO bioavailability also shifts the balance toward a pro-inflammatory, pro-fibrotic state. Normally, NO inhibits leukocyte adhesion and the expression of pro-inflammatory mediators [239]; its reduction contributes to increased inflammation [240]. In the hypertensive heart, mechanical stress, Ang II, and ROS recruit inflammatory cells that release cytokines and growth factors, like TGF-β, stimulating fibroblast activation and extracellular matrix deposition [241,242]. The imbalance between matrix metalloproteinases and their inhibitors further promotes fibrosis, with the NO/cGMP/PKG pathway ordinarily counteracting these effects [241,242]. Consequently, the loss of NO removes an essential inhibitory signal for inflammation and fibroblast activation, leading to maladaptive myocardial fibrosis [235].

#### 3.3.1. Animal Studies

In AH, eNOS (−/−) mice exhibit reduced arteriolar density, lower Ca^2+^ levels within the sarcoplasmic reticulum, and a predominance of K^+^ channels in ventricular cardiomyocytes [243,244]. eNOS uncoupling has been identified as a cause of AH in ANG II-challenged mice [245]. Supplementation with tetrahydrobiopterin (BH4) and vitamin C can prevent eNOS uncoupling by reducing the production of reactive oxygen species, thereby supporting endothelial function in AH [246,247]. eNOS uncoupling and superoxide production have been observed in the myocardium of mice subjected to transverse aortic constriction, a widely used surgical model for studying pressure-overload-induced cardiac hypertrophy and HF [248]. eNOS expression in the myocardium plays a crucial cardioprotective role by mediating vasodilation and neoangiogenesis, maintaining sympathovagal balance, and contributing to myocardial remodeling, which helps prevent severe hypertrophy [249]. Additionally, inhibition of NOS by nitro-L-arginine induces AH in rats and increases myocardial oxidative stress [250].

Hypoxia induces an increase in vascular nNOS expression and activity through enhanced translational efficiency; however, whether this mechanism applies to hypertensive myocardium remains under investigation [251]. ANG II acts as a trigger for the upregulation of nNOS expression and activity in isolated left ventricular cardiomyocytes by reducing NADPH oxidase activity, thereby promoting myocardial relaxation [252]. Furthermore, nNOS expression is upregulated in the left ventricular myocardium of ANG II-induced hypertensive rats [252]. In SHRs, nNOS expression in the myocardium appears to be higher in the LV compared to the RV [79].

The upregulation of iNOS is associated with excessive NO production and may play a significant role in the development of AH. iNOS-derived NO can interact with superoxide anions, promoting the formation of peroxynitrite, which leads to severe endothelial dysfunction and substantial nitrosative stress. Furthermore, increased iNOS activity may reduce eNOS-derived NO, resulting in eNOS uncoupling and the production of primary oxygen radicals. iNOS inhibition has notable antihypertensive effects, contributing to the maintenance of vascular integrity and function [253]. Studies on iNOS (−/−) mice compared to wild-type controls show no significant differences in systolic blood pressure or cardiac structure, suggesting that iNOS may not play a direct role in maintaining local cardiac hemodynamics or morphology [254]. However, the administration of L-NAME in iNOS (−/−) mice is associated with a milder development of cardiac hypertrophy [254]. While the ablation of iNOS does not appear to affect the degree of myocardial hypertrophy, it does mitigate cardiac contractile dysfunction observed in chronic AH [255].

The deletion of iNOS does not influence the severity of two-kidney, one-clip-induced hypertension or myocardial hypertrophy, but it improves cardiac function, potentially due to a reduction in oxidative stress [256]. In iNOS (−/−) mice, chronic transverse aortic constriction is associated with mild myocardial hypertrophy and fibrosis [257]. Excessive upregulation of iNOS expression significantly increases blood pressure and promotes sympathoexcitation through oxidative-stress-mediated mechanisms [258]. However, iNOS-derived NO has been shown to improve salt-sensitive hypertension [259]. The overproduction of NO by iNOS in AH contributes to nitrosative stress and endothelial dysfunction [260]. Administration of pyrrolidinedithiocarbamate, an antioxidant and nuclear factor kappa-B inhibitor, along with aminoguanidine, a selective iNOS inhibitor, significantly attenuates the development of AH and enhances acetylcholine-mediated vascular responses in SHRs [261]. This underscores the protective role of iNOS and its potential involvement in compensatory mechanisms that help prevent the development and progression of AH.

#### 3.3.2. Human Studies

eNOS-derived NO plays a crucial role in maintaining blood pressure by promoting the relaxation of vascular smooth muscle cells through the activation of cGMP-dependent protein kinase. Evidence suggests a deficient vasodilatory effect of NO in the brachial, renal, and coronary arteries, as well as impaired L-arginine transport, in patients with EH and normotensive individuals with a family history of the condition [262]. In hypertensive patients, L-arginine supplementation appears to improve endothelial function via various mechanisms, including altered catecholamine and aldosterone release and modified renin–angiotensin II activity [263]. Overall NO production is reduced in patients with EH, as indicated by lower urinary and plasma nitrate levels [264]. Individuals with a family history of EH exhibit a diminished response to acetylcholine due to alterations in the NO pathway [265]. Furthermore, significant associations have been demonstrated between eNOS gene polymorphisms, such as the Glu298Asp variant, and EH [266]. Additionally, eNOS intron 4a/b gene polymorphisms are implicated in the etiology of EH [267].

nNOS also plays a prominent role in maintaining systemic vascular resistance and, consequently, blood pressure under physiological conditions [228]. nNOS activity is linked to increased production of H_2_O_2_ and NO, contributing to the normalization of blood pressure in hypertensive patients [268]. Hypertensive individuals typically exhibit decreased NO levels, elevated free radical production, and increased oxidative stress [269]. Alterations in nNOS expression and activity may contribute to the development of EH and stress-induced cardiovascular disorders [270]. nNOS-derived NO counteracts ANG II-mediated vascular contractility; however, this antagonistic effect diminishes under hypertensive conditions due to increased oxidative stress and reduced nNOS activity [271,272]. A comparison of human and animal studies on NOS signaling is presented in Table 11.

#### 3.3.3. Potential Therapeutic Implications

A variety of approaches have been explored or are under investigation to enhance NO signaling in cardiovascular disease. One major category includes NO donors, which are drugs that release NO directly in the body. Organic nitrates, such as isosorbide dinitrate (ISDN), have been used clinically, primarily for angina. In heart failure, especially among African American patients, the combination of hydralazine and ISDN (H-ISDN) has demonstrated mortality benefits by reducing cardiac preload and afterload [273]. However, continuous use can lead to tolerance and side effects like headache and dizziness, limiting long-term efficacy [106].

Sodium nitroprusside (SNP) is a potent intravenous vasodilator that spontaneously releases NO, activating the sGC-cGMP pathway. It is effective for hypertensive emergencies and during surgery, though its very short half-life necessitates continuous infusion and it carries a risk of cyanide toxicity if used long term [237].

To overcome the limitations of traditional NO donors, research is developing novel NO delivery systems, including biomaterial-based platforms for sustained and controlled release at target tissues, and metal–nitrosyl complexes that release NO under physiological triggers, with aims to improve efficacy, duration, and safety [237].

eNOS uncoupling plays a pivotal role in the development of AH, making mechanisms that restore eNOS coupling activity particularly relevant in the context of antihypertensive therapy. However, the effects of ANG II on eNOS remain controversial. Some studies indicate that ANG II does not affect eNOS expression or may even reduce it under hypertensive conditions [274]. Tambascia et al., 2001, described increased myocardial eNOS expression in ANG II-infused rats [275]. On the other hand, BH_4_ supplementation attenuates significantly endothelial dysfunction as it stimulates endothelium-dependent vasodilatation in both normotensive and hypertensive subjects [276,277]. BH4 supplementation, or its precursor sepiapterin, aims to restore eNOS coupling, increasing NO and reducing superoxide generation. Preclinical studies show BH4 can reverse hypertrophy and fibrosis, improve cardiac function, and reduce oxidative stress in models of pressure-overload-induced hypertrophy and HHD [234,278]. However, one study suggests these benefits may also involve anti-inflammatory effects, not just NOS recoupling [279]. Clinical translation is limited by BH4′s poor bioavailability and rapid in vivo oxidation [279]. Efforts to enhance endogenous eNOS activity include using statins, which not only lower lipids but also increase eNOS expression and activity via Akt phosphorylation and stabilize the enzyme, boosting NO production [234]. ACEi and ARBs also benefit the NO system by reducing Ang II and oxidative stress, preserving BH4 and enhancing NO bioavailability. Some ACEi may even directly potentiate NO [240].

Another strategy is substrate/cofactor supplementation, enhancing endogenous NO production by providing the necessary elements for NOS enzymes. L-arginine supplementation has been studied in conditions such as hypertension and endothelial dysfunction, but results have been inconsistent [234]. This may be due to the “arginine paradox”, where supplementation fails to increase NO production despite high intracellular L-arginine levels. Factors like impaired uptake, increased arginase activity, or presence of inhibitors like ADMA may limit effectiveness [234]. Altered L-arginine transport has been observed in both normotensive and hypertensive patients with a genetic predisposition to EH [261]. L-arginine supplementation is associated with a marked reduction in endothelial dysfunction in AH [262]. Similarly, the intake of NOS substrates such as L-arginine or its precursor, L-citrulline, demonstrates promising antihypertensive effects in rat models of AH [280]. Vitamin C plays a crucial role in maintaining BH4 bioavailability during vascular oxidative stress and prevents eNOS uncoupling [281,282,283]. BH4 supplementation reduces eNOS-dependent reactive oxygen species production while increasing NO generation in SHRs [284]. However, the therapeutic potential of BH4 as an antihypertensive agent is limited by its chemical instability. Sepiapterin, a compound involved in BH4 metabolism and a potential therapy for phenylketonuria, can be converted to BH4 via the biopterin salvage pathway and may offer potential in antihypertensive treatment [285]. Indeed, sepiapterin administration significantly enhances endothelium-dependent vasodilation [286].

Additionally, nitrite administration has been shown to maintain myocardial function and lower blood pressure in hypertensive patients [287]. Selective nNOS inhibition in humans results in a dose-dependent increase in mean arterial and diastolic blood pressure, accompanied by a decrease in heart rate [228]. Oxidative stress contributes to the development of AH and leads to compensatory upregulation of eNOS and iNOS expression in SHRs. Antioxidant administration has been shown to improve AH by attenuating the overexpression of NOS isoforms [288]. Moreover, the use of iNOS inhibitors reduces blood pressure, suggesting that iNOS could serve as a potential target for pharmacological treatment of AH [253]. Treatment of hypertensive patients with lacidipine, a calcium channel blocker, has demonstrated significant reductions in plasma lipid peroxidation and macrophage iNOS activity, thereby decreasing free radical formation and preserving endothelial function [210]. Specific eNOS activators, such as AVE3085, are being developed. In preclinical models of diastolic heart failure, AVE3085 increased eNOS expression and activity, improved NO production, reduced oxidative stress, and mitigated diastolic dysfunction, hypertrophy, and fibrosis [289].

Another promising direction targets downstream signaling, specifically the NO-activated soluble guanylate cyclase (sGC). sGC stimulators like riociguat enhance the enzyme’s response to endogenous NO, while sGC activators like cinaciguat and vericiguat activate it independently of NO [237]. Riociguat is approved for pulmonary arterial hypertension [273], and vericiguat has shown benefits in reducing hospitalizations in HFrEF patients [273]. Since cGMP signaling is impaired in HFpEF, these agents may offer future treatment potential [241].

Phosphodiesterase (PDE) inhibitors, particularly PDE5 inhibitors such as sildenafil and tadalafil, prevent cGMP degradation, prolonging NO’s effects [232,235]. These drugs are approved for PAH [273] and erectile dysfunction. However, clinical trials in HF, especially HFpEF, have been largely inconclusive or disappointing [273].

Dietary strategies also play a role. Inorganic nitrate/nitrite, found in foods like beetroot and leafy greens, can be converted into NO via the enterosalivary nitrate–nitrite–NO pathway, especially under hypoxic or acidic conditions [227]. This provides an alternative NO source, independent of NOS. Preclinical studies have shown nitrate supplementation reduces blood pressure and cardiac remodeling, and acute clinical trials suggest potential benefits for exercise capacity in heart failure patients, although long-term effects remain uncertain [227,273].

Given the unique and sometimes opposing roles of the NOS isoforms, developing isoform-selective therapies is a key goal [290].

nNOS modulation has been studied using selective inhibitors like S-methyl-L-thiocitrulline (SMTC). In healthy humans, SMTC increased systemic vascular resistance and blood pressure, confirming nNOS’s role in regulating vascular tone [228]. However, therapeutic use in HHD is unclear since nNOS may also have protective roles (e.g., anti-hypertrophic effects [227], central sympathoinhibition [230]). Selective nNOS activation may be beneficial but must be approached cautiously due to its diverse roles.

iNOS modulation is more established in inflammatory diseases. Selective iNOS inhibitors have been explored for conditions like sepsis or arthritis [290]. Their use in chronic HHD or heart failure is still being investigated, with a focus on reducing harmful NO levels without compromising immune function or other NOS activities.

eNOS modulation remains a priority in HHD. Enhancing eNOS activity—via BH4 supplementation or compounds like AVE3085—has shown promise in improving endothelial function and cardiovascular outcomes [278].

Targeting specific NOS isoforms allows for more precise therapy, potentially avoiding side effects of broad NOS inhibition or activation. However, achieving high selectivity and understanding complex interactions among isoforms in various disease states remain ongoing challenges [291]. Table 12 compares the available therapies with possible future approaches concerning all the discussed molecules.

**Figure 4:**
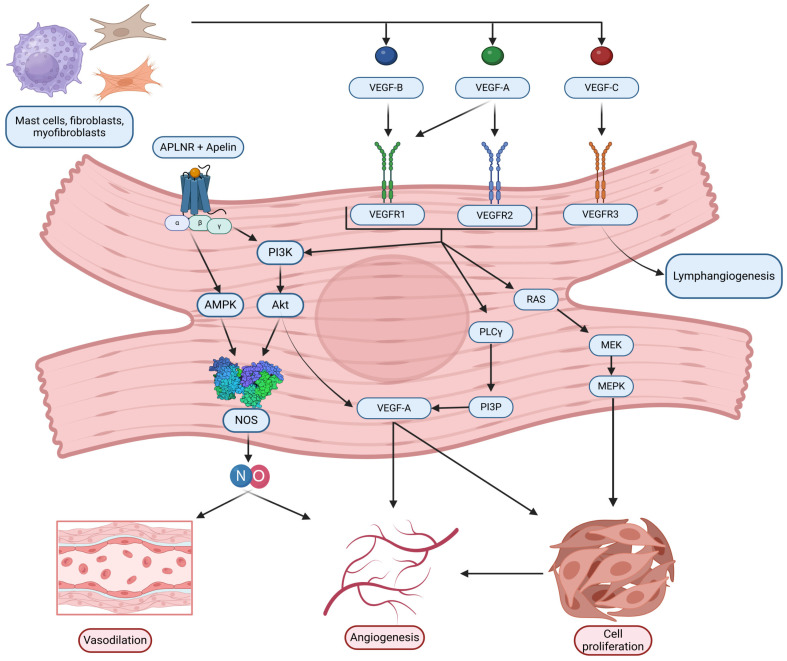
Graphical representation of the interplay between apelin/apelin receptor (APLNR), vascular endothelial growth factor (VEGF)/VEGF receptors (VEGFR), nitric oxide (NO)/nitric oxide synthase (NOS) signaling pathways in cardiomyocytes in the context of hypertensive heart disease. Original figure created with BioRender.com.

The intricate interplay between the apelinergic system, VEGF/VEGFR, and NO/NOS signaling pathways in cardiomyocytes in the context of hypertensive heart disease is summarized and visually represented in Figure 4. The figure also illustrates the connections between mast cells, fibroblasts, and myofibroblasts with the discussed molecular pathways and their roles in angiogenesis and CD changes during myocardial remodeling.

## 4. Limitations

This review paper has several limitations that should be acknowledged to provide context for its conclusions:

1. Potential for publication bias: Like many reviews, this paper might be susceptible to publication bias, where studies showing positive or significant results are more likely to be published and included than those with negative or inconclusive findings. This could skew the overall picture presented for some pathways or therapeutic interventions.
2. Scope of Studies: While this review extensively covers the apelinergic system, VEGF/VEGFR pathway, and NO/NOS signaling, it predominantly relies on animal studies, particularly those involving SHRs. The generalizability of these findings to humans might be constrained by interspecies differences in cardiovascular physiology and pathology.
3. Human data gaps: Despite the inclusion of some human studies, there is a notable disparity in the depth and quantity of research compared to animal studies. For example, the role of mast cells and VEGF dynamics in human hypertensive myocardium remains underexplored, which is potentially limiting the applicability of findings to clinical settings.
4. Variability in study design: The studies reviewed demonstrate considerable variability in experimental designs, methodologies, and endpoints, complicating direct comparisons and synthesis of findings. This heterogeneity may introduce bias and limit the ability to draw definitive conclusions.
5. Therapeutic context: While potential therapeutic targets are discussed, the translation of these findings into clinical interventions is still in preliminary stages. The long-term safety and efficacy of proposed treatments, such as apelin analogs and VEGF modulation, require further clinical validation.

These limitations highlight the need for more comprehensive and translational research, particularly focusing on human studies and clinical trials, to validate the therapeutic potential of the pathways and targets discussed. The reliance on animal models, particularly SHRs, can be countered by increasing studies in diverse human populations, utilizing a wider range of animal models when necessary, and employing human-relevant in vitro systems. Specific gaps in human data require targeted investigation using patient samples and advanced imaging. Variability in study designs necessitates the promotion of standardized protocols and reporting guidelines, alongside rigorous methods for evidence synthesis. Finally, the preliminary nature of therapeutic translation calls for large-scale, long-term randomized clinical trials focused on patient-relevant outcomes to validate the safety and efficacy of proposed treatments like apelin analogs or pathway modulators before clinical adoption.

## 5. Conclusions

This review comprehensively highlights the intricate molecular and cellular mechanisms underlying hypertensive myocardium, emphasizing the role of capillary density, fibrosis, mast cells, the apelinergic system, VEGF/VEGFR pathways, and NO/NOS signaling, in the progression of hypertension-induced cardiac remodeling. These mechanisms operate synergistically, driving structural and functional changes in the heart, including left ventricular hypertrophy, fibrosis, impaired angiogenesis, and diastolic dysfunction.

Capillary rarefaction and myocardial fibrosis are hallmark features of HHD. While animal models provide valuable insights into these processes, human studies remain limited. Targeting fibrosis through RAAS inhibitors, anti-inflammatory agents, or novel anti-fibrotic therapies could potentially improve outcomes in hypertensive patients. Mast cells, though less studied in human hypertensive myocardium, play a crucial role in cardiac remodeling by releasing pro-fibrotic mediators such as FGF-2 and histamine. Targeting mast cell activity represents a potential, albeit underexplored, avenue for therapeutic intervention.

The apelinergic system emerges as a promising therapeutic target due to its cardioprotective effects, including vasodilation, anti-fibrotic properties, and the ability to counteract the RAAS. Studies demonstrate its potential in mitigating ANG II-mediated cardiac remodeling and preserving myocardial function. However, its therapeutic utility in humans remains underexplored, with further research needed to optimize apelin analogs and assess their long-term efficacy in clinical settings.

Similarly, VEGF signaling plays a dual role, supporting compensatory angiogenesis in the early stages of hypertensive remodeling but contributing to adverse outcomes when dysregulated. Enhancing VEGF bioavailability, either through exercise or pharmacological interventions, shows promise in mitigating maladaptive cardiac changes. Conversely, VEGF inhibitors, commonly used in oncology, can exacerbate AH and endothelial dysfunction, underscoring the need for careful therapeutic balancing.

The NO/NOS signaling pathway is pivotal in maintaining vascular tone, promoting angiogenesis, and preventing oxidative stress. However, eNOS uncoupling and iNOS overexpression in AH disrupt this balance, leading to endothelial dysfunction and nitrosative stress. Restoring NO bioavailability through BH_4_ supplementation, antioxidants, or L-arginine holds potential but requires further validation in human studies.

Despite significant advances, several gaps remain in the current understanding of hypertensive myocardium. The reliance on animal models and limited human studies restricts the translational applicability of findings. Furthermore, variability in study designs and selective focus on specific pathways limit the comprehensive understanding of the disease’s multifactorial nature.

Future research should prioritize human studies and clinical trials to validate the therapeutic potential of the discussed pathways. A multidisciplinary approach integrating molecular insights, clinical observations, and innovative therapies could pave the way for more effective and personalized treatments for HHD. Addressing these challenges will not only enhance our understanding of the disease but also improve the prognosis and quality of life for patients with hypertension.

## Figures and Tables

**Table 1:** Comparison of animal and human studies on capillary density (CD).

Feature	Animal Studies	Human Studies
**Models/Subjects**	**Models Used:** Spontaneously hypertensive rats (SHRs) vs. normotensive Wistar-Kyoto (WKY) rats, as well as other models (Dahl salt-sensitive rats; C57BL/6J mice on a high-salt/ANG II regimen).	**Subjects:** Adults aged 19–55 years, categorized as normotensive, untreated hypertensive, and hypertensive under therapy. Biopsy samples from patients with hypertensive heart disease (HHD) were also analyzed.
**Method of Assessment**	**Measurements:** Direct evaluation of CD in heart tissue (e.g., left and right ventricles) and capillary-to-fiber ratio.	**Measurements:** CD assessed indirectly via nailfold skin analysis (functional and structural assessments) and directly via biopsy samples in some studies.
**Key Findings on Capillary Density**	**Increased CD:** Some studies report increased CD in SHRs (often associated with elevated VEGF expression and a higher capillary-to-fiber ratio) observed even in young rats (e.g., 4-week-old), suggesting early compensatory vascular remodeling. •**Decreased CD:** Other studies (e.g., Caudron et al.) report decreased CD, particularly in older SHRs with severe hypertension-induced cardiac injury, with similar rarefaction seen in other hypertensive models.	**Capillary Rarefaction:** Some studies (e.g., Cheng et al. and Prewitt et al.) indicate an initial functional rarefaction followed by irreversible structural rarefaction without significant differences in structural CD in certain assessments. - Others (e.g., Penna et al. and biopsy studies in HHD patients) report significant structural deficits (up to ~25% lower CD).
**Timing of Changes**	Increased CD observed before significant changes in blood pressure in some studies. Decreased CD associated with later stages of hypertension and cardiac injury in other studies.	Functional rarefaction may precede structural rarefaction. Pronounced structural rarefaction evident in established hypertensive heart disease.
**Proposed Mechanism(s)**	**Increased CD:** Compensatory mechanism for hypoxia, linked to elevated VEGF. **Decreased CD:** Linked to reduced VEGF expression, contributing to hypertension-induced cardiac injury.	**Functional Rarefaction:** Initial stage. **Structural Rarefaction:** Later stage, potentially irreversible, contributing to cardiac injury. Reduced vascular network in HHD impacting heart function.
**Implications**	**Early vs. Late Remodeling:** The findings suggest that early increases in CD (possibly as a compensatory mechanism) may precede the development of overt hypertension, whereas later decreases in CD (capillary rarefaction) may contribute to hypertension-induced cardiac injury.	**Vascular Health:** The structural and functional capillary rarefaction observed in hypertensive individuals underlines the importance of the vascular network in maintaining proper heart function and may be key in understanding the progression of hypertensive cardiac injury

**Table 2:** Collagen alterations in hypertensive heart disease.

Parameter	Change in HHD	Key Mechanisms/Mediators	Functional Consequence
**Total Collagen Content**	Increased (interstitial and perivascular fibrosis)	Increased fibroblast/myofibroblast activity, increased synthesis leading to decreased degradation; stimuli: pressure overload, RAAS, TGF-β	Increased myocardial stiffness, diastolic dysfunction, arrhythmogenesis
**Collagen Type I**	Increased deposition	Preferential synthesis/deposition by myofibroblasts	Highly increased myocardial stiffness (major contributor), diastolic dysfunction
**Collagen Type III**	Increased deposition (but less proportionally than type I)	Synthesis by myofibroblasts	Contributes to fibrosis, but less impact on stiffness than type I
**Type I/III Ratio**	Increased	Disproportionate increase in type I synthesis/deposition	Highly increased myocardial stiffness, reduced compliance, diastolic dysfunction

**Table 3:** Key signaling pathways implicated in HHD-associated fibrosis.

Pathway Name	Key Stimuli in HHD	Key Mediators/Receptors	Primary Effect on Fibroblasts and ECM	Key Downstream Effectors
**Mechanical stress**	Increased pressure overload and cardiac wall stress, tissue stiffness	Integrins, mechanosensitive ion channels	Activation, differentiation, elevated collagen synthesis	FAK, MAPKs, RhoA/ROCK, YAP/TAZ
**Angiotensin II**	Pressure overload, sympathetic nervous system activation, local production	ANG II, AT1R	Proliferation, differentiation into myofibroblasts, elevated ECM synthesis, elevated TGF-β	MAPK, SMADs, PLC
**Aldosterone**	AngII stimulation	Aldosterone, Mineralocorticoid Receptor (MR)	Fibroblast activation, elevated collagen synthesis and oxidative stress, inflammation	MR nuclear translocation, MAPK
**TGF-β**	Ang II, mechanical stress, inflammation, FGF23	TGF-β1, TGF-β Receptors (Type I/II)	Differentiation (myoFb), ↑Collagen I/III synthesis, ↑Fibronectin, ↑TIMP expression	SMAD2/3 phosphorylation and nuclear translocation

**Table 4:** Comparison of animal and human studies on fibrosis.

Feature	Animal Studies	Human Studies
**Models/Subjects**	Spontaneously hypertensive rats (SHRs) compared to normotensive WKY rats.	Hypertensive patients.
**Fibrosis Type**	Predominantly reactive fibrosis characterized by increased interstitial collagen deposition.	Four histologically distinct types: interstitial, compact, diffuse, and patchy, with a common feature of increased collagen deposition.
**Cardiac Changes**	Increased LV and RV wall thickness. Elevated collagen deposition in the cardiac interstitium, more pronounced in the LV. Severity increases with age and progression of HHD.	Increased deposition of collagen types I and III in various patterns (interstitial, compact, diffuse, patchy). Increased LV wall thickness observed in correlation with elevated TGF-β.
**Collagen Composition**	Excessive deposition of collagen types I and III. Normal or downregulated collagen degradation by matrix metalloproteinases.	Collagen type I is the predominant type, followed by collagen type III.
**Age-Related Changes**	More pronounced fibrosis in 12-month-old SHRs (advanced hypertensive heart disease) compared to 6-month-old SHRs (early AH development).	Although specific age-related dynamics are not detailed, increased fibrosis correlates with higher LV wall thickness and elevated levels of profibrotic factors such as TGF-β.
**Key Signaling Pathways and Markers**	FGF-2 Pathway: moderate increase in FGF-2 expression, correlating with regions of enhanced fibrosis. •Procollagen type I mRNA: elevated in the LV. •ANG II: administration increases blood pressure and fibrosis, linked with higher TGF-β levels.	Fibroblast activation: increased transformation of fibroblasts into myofibroblasts drives collagen production. •Inflammatory response: inflammation elevates TGF-β levels, correlating with increased LV wall thickness. •ANG II: elevated levels observed, with evidence that ANG II blockade reduces fibrotic changes.
**Cellular Mechanisms**	Increased collagen production likely due to fibroblast activation.	Transformation of fibroblasts into myofibroblasts drives ECM production, including collagen. Myofibroblast transformation and activation are exacerbated by inflammation.
**Role of Inflammation**	Not explicitly detailed in the text regarding direct inflammatory involvement in SHRs, but other pathways (like ANG II/TGF-β) have inflammatory components.	Fibrotic changes are closely linked with inflammation, which exacerbates collagen deposition through increased myofibroblast transformation and activation. Immunocompetent cells at inflammatory sites increase TGF-β levels.
**Therapeutic Implications**	ANG II blockade shown to reduce fibrosis in SHRs, suggesting a potential therapeutic target.	Studies using ANG II blockade have demonstrated reduced fibrotic changes in the heart, highlighting its potential therapeutic role.

**Table 5:** Key cardiac mast cell mediators and their implicated roles in HHD pathophysiology.

Mediator	Source (Preformed/Synthesized)	Key Implicated Actions in HHD
**Chymase**	Preformed	Fibrosis promotion (TGF-β activation, fibroblast proliferation, collagen synthesis, procollagen processing); ACE-independent Ang II formation; cardiomyocyte apoptosis; MMP activation; potential ECM degradation
**Tryptase**	Preformed	Fibrosis promotion (fibroblast proliferation/activation via PAR-2); MMP activation; potential ECM degradation; HDL degradation
**Histamine**	Preformed	Increased vascular permeability; potential fibrosis role (H2R?); endothelial dysfunction; vasodilation/constriction (context-dependent); Prostaglandin release from fibroblasts
**TNF-α**	Preformed/Synthesized	Pro-inflammatory; pro-hypertrophic; pro-fibrotic (via AT1R upregulation); MMP activation; cardiac dysfunction
**IL-6**	Synthesized	Pro-inflammatory; endothelial dysfunction (via ROS, ↓p-eNOS in IgE pathway); potential hypertrophy role
**Renin**	Preformed	Initiates local cardiac RAS → Ang II formation
**TGF-β**	Synthesized (also activated by Chymase)	Potent pro-fibrotic (fibroblast activation, collagen synthesis); potential pro-hypertrophic
**VEGF**	Synthesized	Angiogenesis; potential protective/reparative role
**IL-10**	Synthesized	Anti-inflammatory; potential anti-fibrotic

**Table 6:** Comparison of animal and human studies on mast.

Feature	Animal Studies	Human Studies
**Evaluation Method in HHD**	Direct measurement of MCN and correlation with fibrosis and specific mediators is feasible and reported.	Evaluation currently relies on assessing the neurochemical profile (mediators) rather than directly quantifying MCN in the context of HHD.
**Mast Cell Numbers (MCN)**	•Statistically significant increased MCN observed in SHRs, with a statistically significant rise from 6- to 12-month-old rats. •Increase correlates with elevated FGF-2 levels and expanded fibrotic areas. - MCN rise seen in both the LV and RV (less pronounced in the RV).	•Increased MCN reported in patients with dilated cardiomyopathy. •Transplanted human heart studies reveal a correlation between cardiac mast cells and interstitial as well as perimyocytic fibrosis. •Direct investigation in hypertensive conditions is lacking.
**FGF-2 Association**	•Mast cells are a significant source of FGF-2, a key profibrotic factor. •FGF-2 expression correlates with increased fibrosis in animal models.	•FGF-2 in humans shows profibrotic effects similar to animal models. •However, there is no direct study linking increased MCN to higher FGF-2 expression in hypertensive heart disease.
**Role of FGF-2**	Mast cells identified as an important source of FGF-2, linking increased MCN to this profibrotic agent.	FGF-2 exhibits the same profibrotic effects (fibroblast activation, etc.) as in animal models. •No studies directly link MCN to FGF-2 expression in human HHD.
**Role of Tryptase**	Demonstrates profibrotic role via PAR-2/MAPK/ERK pathway, driving fibroblast-to-myofibroblast transformation and collagen synthesis. •Increases cell proliferation and collagen I synthesis in rats.	Tryptase exhibits the same profibrotic effects as in animal models. •No studies directly link MCN to tryptase activity/expression in human HHD.
**Role of Chymase**	Shows profibrotic effects (e.g., TGF-β activation). •Generates ANG II, contributing to proinflammatory and profibrotic changes.	Chymase exhibits the same profibrotic effects (fibroblast activation, inflammation, ANG II generation) as in animal models. •No studies directly link MCN to chymase activity/expression in human HHD.
**Role of Histamine**	Elevated histamine levels and increased H2R expression found in SHRs compared to normotensive rats, linked to increased MCN.	Histamine exhibits the same profibrotic effects as in animal models. •No studies directly link MCN to histamine levels/activity in human HHD.
**Mechanisms of Fibroblast Activation**	Mast cell-derived mediators (tryptase, chymase, histamine) contribute to fibroblast activation and myofibroblast transformation via specific signaling pathways (e.g., PAR-2 → MAPK/ERK). •Mast cells may release both antifibrotic and profibrotic mediators depending on microenvironmental signals.	The profibrotic roles of FGF-2, tryptase, chymase, and histamine are acknowledged based on their neurochemical actions. •Direct mechanistic links between mast cell numbers and fibroblast activation under hypertensive conditions in humans remain to be elucidated.
**Complexity of Role**	Noted that mast cells can release both pro- and anti-fibrotic mediators depending on the microenvironment.	Primarily emphasizes the profibrotic roles of known mediators.

**Table 7:** Mast-cell-targeted therapeutic strategies: preclinical and clinical landscape.

Therapeutic Strategy	Specific Agent(s)/Approach	Key Preclinical Findings in CV Models (HHD, HF, Fibrosis)	Clinical Trial Status/Notes
**Mast Cell Stabilization**	Tranilast, Nedocromil Sodium, Disodium Cromoglycate	Prevented HF transition; reduced fibrosis (pressure overload, SHR models)	Primarily used/trialed for allergy/mastocytosis; no large CV trials for HHD/HF.
**εPKC Inhibition**	εV1-2 peptide inhibitor	Attenuated HF progression, reduced fibrosis, prevented MC degranulation (hypertensive HF model)	Preclinical stage for this mechanism.
**Chymase Inhibition**	Various specific inhibitors (e.g., Chymostatin in preclinical)	Reduced fibrosis, improved diastolic function, reduced Ang II/TGF-β activation (HF, pressure overload models)	Preclinical validation strong; potential to address residual risk; clinical CV trials needed.
**Tryptase Inhibition**	Specific inhibitors	Rationale based on pro-fibrotic effects; serum tryptase as potential biomarker?	Inhibitors exist; clinical CV trials needed.
**Histamine Receptor Antagonism**	H2 Receptor Antagonists (e.g., Famotidine)	Associated with reduced HF risk (observational human data); decreased infarct size (canine ischemia model)	Requires prospective RCT validation for CV indications.
**Anti-IgE Therapy**	Omalizumab	Attenuated Ang II-induced hypertension and vascular remodeling (mouse model); alleviated HF/remodeling (mouse HF models)	Approved for allergy/asthma; CV effects largely unexplored; some trials exclude severe CV disease.
**Anti-IL-6 Therapy**	IL-6 inhibitors (e.g., Tocilizumab)	Rationale based on IgE-MC-IL-6 pathway in hypertension/endothelial dysfunction.	Approved for inflammatory diseases; potential CV relevance via this pathway needs investigation.

**Table 8:** Comparison of animal and human studies on apelinergic system.

Feature	Animal Studies	Human Studies
Model/Subjects	•Spontaneously hypertensive rats (SHRs). •Two-kidney, one-clip (2K1C) hypertensive rats. •Hypertensive rats induced by L-NAME. •Apelin-knockout mice.	•Essential hypertension (EH) patients. •Untreated hypertensive patients. •Patients with acute coronary syndrome (ACS). •Healthy controls.
Focus	Hypertension-induced cardiac remodeling, mechanisms of apelin/APLNR in HHD.	Association of apelin levels with LVH, blood pressure regulation, and cardiovascular risk in hypertensive patients.
Role in Cardiac Remodeling	The apelin/APLNR system regulates myocardial hypertrophy, fibrosis, vascular function, and neurohormonal interactions. •Acts as a compensatory mechanism in response to ligand depletion in advanced AH.	•Reduced serum apelin levels are strongly associated with left ventricular hypertrophy (LVH) in essential hypertension (EH). •Implicated in blood pressure regulation and pathological cardiac remodeling.
Key Findings (Apelin/APLNR Expression)	•Upregulation of APLNR in aged SHRs (compensatory mechanism). •Dynamic APLNR changes in 2K1C rats (reduced in acute, partial recovery in chronic). •Differential organ-specific apelin/APLNR expression (increased in cardiac, decreased in renal). •Increased apelin in the rostral ventrolateral medulla of SHRs (sympathetic overdrive). •Reduced apelin levels in hypertensive rats with LVH.	•Low serum apelin levels associated with LVH in essential hypertension (EH). •Lower apelin levels in untreated hypertensive patients. •Increased apelin levels with antihypertensive treatment. •Lower apelin levels in EH and acute coronary syndrome (ACS) patients, especially females.
Key Findings (Functional Effects)	•Apelin administration reduces blood pressure and LV systolic pressure. •Apelin mitigates ANG II-induced cardiac fibrosis, hypertrophy, and dysfunction. —Apelin-knockout exacerbates cardiac damage under ANG II exposure. •Apelin-normalized hypertension induced heterodimerization of APLNR and kappa-opioid receptor.	•Reduced apelin levels associated with LVH and blood pressure dysregulation. •Apelin levels may be a biomarker for AH management.
Expression Dynamics	•APLNR Expression: Upregulated in aged SHRs, suggesting compensation. •In 2K1C hypertensive rats, myocardial APLNR levels are reduced in the acute phase with partial recovery in the chronic phase, while aortic APLNR mRNA declines consistently, with protein reduction evident in the chronic phase. •Differential responses observed: marked reduction in apelin/APLNR in SHRs vs. only mild changes in 2K1C models, and tissue-specific alterations (e.g., increased in cardiac tissue, reduced in kidney tissue in L-NAME-induced hypertension).	•Apelin levels: lower in untreated hypertensive patients compared to normotensive controls. •Antihypertensive treatment increases apelin levels, indicating its regulatory role. •Genetic studies show significantly lower apelin-13 levels in EH and acute coronary syndrome patients, with possible sex-specific effects (e.g., risk allele in women).
Cardiovascular Effects	•Functional effects: Apelin administration reduces mean arterial pressure and left ventricular systolic pressure. •Mitigates ANG II-induced cardiac fibrosis, hypertrophy, and dysfunction. •Increased apelin in specific brain regions (rostral ventrolateral medulla) contributes to sympathetic overdrive.	•Lower apelin levels correlate with LVH and increased cardiovascular risk. •Suggested potential as a biomarker for disease severity and response to antihypertensive therapy.

**Table 9:** Therapeutic implications of the apelinergic system.

Compound Name/Type	Mechanism	Key Preclinical/Clinical Findings (HHD/HF Relevant)	Status/Potential
[Pyr1]apelin-13 (Native Peptide)	Full Agonist	Acute ↑CO, ↓PVR, ↓BP, +inotropy; Preserved in HF/PAH; chronic infusion failed to ↓BP or prevent AngII-induced hypertrophy/fibrosis.	Proof-of-concept; limited by short half-life.
Elabela (Native Peptide)	Agonist	↓BP, +inotropy, anti-fibrotic, anti-remodeling; Beneficial in hypertension/HF models; Critical for development.	Potential therapeutic; stability/delivery challenges similar to apelin.
BMS-986224 (Small Molecule)	Agonist (Non-biased)	Potent APJ activation; ↑CO in rats (acute/chronic); did not prevent hypertrophy/fibrosis in RHR model.	Oral bioavailability; phase 1 trials initiated (some terminated/completed). Potential for HF.
MM07 (Peptide Analogue)	Biased Agonist (G protein-preferred)	Enhanced/sustained vasodilation vs. apelin-13 (human); ↑CO (rodent); reduced desensitization; attenuated experimental PAH features.	Potential for improved efficacy/reduced side effects (hypertrophy); early clinical validation.
Azelaprag (AMG 986/BGE-105) (Small Molecule)	Agonist	Potent agonist (pEC50 ~9.5).	Potential oral therapeutic; further data needed from snippets.
CMF-019 (Small Molecule)	Biased Agonist (G protein-preferred)	Potent biased agonist (pEC50 ~10.0).	Potential oral biased therapeutic; further data needed from snippets.

**Table 10:** Comparison of animal and human studies on VEGF/VEGFR signaling.

Feature	Animal Studies	Human Studies
Overall Role of VEGF/VEGFR	•Central role in angiogenesis, cardiac remodeling, and transition from adaptive hypertrophy to Heart Failure (HF). •Inhibition of VEGF accelerates the transition to HF.	•Multifaceted role: promotes adaptive myocardial angiogenesis •Dysregulated signaling linked to endothelial dysfunction and cardiovascular risk.
General Role	Central to mediating angiogenesis, adaptive remodeling, and the transition from compensatory hypertrophy to heart failure (HF).	Multifaceted role balancing adaptive angiogenesis and maladaptive endothelial dysfunction; linked to both protective and pathological responses.
VEGF Expression Dynamics	Induced during compensatory hypertrophy; levels fluctuate with age/stage (e.g., initial increase, later decline in SHRs) •Significant increase noted at specific stages (e.g., 5-fold VEGF-A in 18-week SHRs) [63]. —Detailed regional changes reported (e.g., LV depletion in older SHRs). •Different isoforms (VEGF188, VEGF-C) show specific patterns.	Elevated XBP1 associated with increased VEGF-A in hypertrophic/failing hearts (suggesting adaptive role) •PGE1 increases VEGF-1 production in cardiac myocytes. •Conflicting findings on plasma VEGF levels: higher levels associated with risk vs. lower levels in hypertensives compared to controls.
VEGF and Angiogenesis/Capillary Density	•VEGF-A upregulation can increase capillary-to-myocyte ratio but may reduce net Capillary Density (CD) due to myocyte growth. •VEGF-A depletion correlates with reduced CD and transition to HF. •VEGFR-2 is central to angiogenesis; VEGFR-1/sVEGFR-1 modulates VEGF availability. •VEGF-C/VEGFR-3 involved in lymphangiogenesis. •TRPV4 deletion enhances VEGF-A/VEGFR2 mediated angiogenesis.	•VEGF-A drives myocardial angiogenesis in hypertrophy. •VEGF-1 promotes endothelial cell proliferation/tube formation. •Lower VEGF activity (via polymorphism rs3025039) inversely correlated with atherosclerosis in some hypertensive patients.
Specific VEGF Isoforms/Family	•Extensive study of VEGF-A dynamics. •VEGF188 studied. •VEGF-B implicated in metabolic adaptation and maladaptive hypertrophy/fibrosis. •VEGF-C linked to lymphangiogenesis under high salt/ANG II stimulation.	•VEGF-A studied in relation to XBP1. •VEGF-1 (esp. VEGF165) induced by PGE1. •Plasma VEGF levels studied (isoform often unspecified). •Genetic polymorphism affecting VEGF activity studied.
Receptor Involvement	•VEGFR-2 is central for angiogenesis, while VEGFR-1 (and its soluble form sVEGFR-1) regulates VEGF bioavailability by sequestering VEGF. •TRPV4 suppression boosts VEGFR-2-mediated angiogenesis. •VEGF-C/VEGFR-3 activation promotes lymphangiogenesis that may impact cardiac edema and remodeling.	•Studies indicate involvement of VEGF receptors indirectly through markers of endothelial function (e.g., associations with von Willebrand factor and soluble Flt-1). •Direct receptor-specific investigations are limited, though the impact on angiogenesis and endothelial function is noted.
Impact on Cardiac Remodeling	•Early VEGF upregulation supports capillary density and compensatory angiogenesis; later depletion of VEGF-A, especially in the LV, correlates with reduced capillary density and progression to HF. •Overexpression of VEGF-B may lead to hypertrophy, fibrosis, and metabolic exhaustion, while VEGF-C/VEGFR-3 influences lymphangiogenesis and edema.	•VEGF-A appears to promote myocardial angiogenesis to mitigate ischemic damage and support adaptation during hypertrophic stress. •However, dysregulated VEGF signaling (with either elevated or reduced levels) is linked with endothelial dysfunction and adverse cardiovascular outcomes.
Regulatory Mechanisms	Complex regulation involves: mechanical stretch (NFκB), XBP1 (UPR), secretin levels, TRPV4 signaling, PlGF (releasing VEGF from sVEGFR-1), ANG II, high salt.	XBP1 linked to VEGF-A regulation. •PGE1 induces VEGF-1 via cAMP pathway.
Link to Endothelial Dysfunction/NO	Implied in models (e.g., reduced NO in secretin KO) and general vascular remodeling context.	Elevated VEGF correlated with markers of endothelial damage (vWf, sFlt-1). •Impaired VEGF-NO relationship observed in untreated hypertensive patients.
Genetic Factors	Primarily studied via knockout/transgenic models (e.g., Secretin KO, VEGFR-1 deficiency, VEGF-B overexpression, TRPV4 KO).	Focus on polymorphisms, e.g., VEGF rs3025039 associated with reduced VEGF activity and potentially lower atherosclerosis risk in specific hypertensive populations.
Volume of Research	Extensive data available from numerous studies exploring various facets of the pathway.	Relatively few studies compared to animal research. •Text explicitly highlights the disparity and the need for more human-focused research.
Research Gaps/Limitations	•Extensive animal data elucidate the dynamic, tissue-specific, and isoform-specific regulation of VEGF/VEGFR signaling in hypertensive remodeling, yet these models may not fully recapitulate human pathophysiology.	•Fewer studies are available; existing data show conflicting trends in VEGF expression. •More research is needed to clarify receptor-specific roles, the interplay with endothelial mediators (e.g., NO, vWf, sFlt-1), and the genetic determinants of VEGF activity in hypertensive myocardium.

**Table 11:** Comparison of animal and human studies on NOS signaling.

Feature	Animal Studies	Human Studies
eNOS Function and Expression	•eNOS is cardioprotective: its expression supports vasodilation, neoangiogenesis, and helps maintain myocardial remodeling, which prevents severe hypertrophy. •eNOS (–/–) mice show reduced arteriolar density, altered Ca^2+^ handling, and changes in K^+^ channel predominance in ventricular cardiomyocytes.	•eNOS-derived NO is critical for relaxing vascular smooth muscle via cGMP-dependent protein kinase, thereby maintaining blood pressure. •Hypertensive patients exhibit deficient NO-mediated vasodilation in the brachial, renal, and coronary arteries, with overall reduced NO production (lower urinary and plasma nitrate levels).
eNOS Uncoupling and Interventions	eNOS uncoupling (observed in ANG II-challenged mice and pressure overload models) leads to superoxide production and contributes to AH. •Supplementation with tetrahydrobiopterin (BH_4_;) and vitamin C can prevent eNOS uncoupling by reducing reactive oxygen species.	Although not directly evaluated by gene deletion models, impaired NO bioavailability in EH patients (and altered L-arginine transport) suggests that eNOS dysfunction plays a role in endothelial dysfunction. L-arginine supplementation improves endothelial function.
nNOS Expression and Role	•Hypoxia increases vascular nNOS expression and activity (though its role in hypertensive myocardium needs further investigation). •ANG II upregulates nNOS in isolated LV cardiomyocytes and in ANG II-induced hypertensive rats; nNOS expression is higher in the LV than in the RV of SHRs. •nNOS contributes to myocardial relaxation via reduced NADPH oxidase activity.	•nNOS helps maintain systemic vascular resistance and blood pressure through NO and H_2_O_2_ production. •In hypertensive patients, alterations in nNOS expression/activity may contribute to diminished counteraction of ANG II-mediated vascular contractility, especially under increased oxidative stress.
iNOS Upregulation and Effects	•Upregulation of iNOS leads to excessive NO production, which can interact with superoxide to form peroxynitrite, thereby promoting nitrosative stress and endothelial dysfunction. •iNOS upregulation may reduce eNOS-derived NO, leading to further oxidative stress. •Genetic ablation or pharmacologic inhibition of iNOS in animal models often improves cardiac contractile function and reduces hypertrophy despite not significantly altering blood pressure.	•In humans, the overproduction of NO via iNOS is implicated in nitrosative stress and endothelial dysfunction in AH. •Pharmacologic interventions that selectively inhibit iNOS (e.g., aminoguanidine) or reduce oxidative stress have shown improvements in vascular responses in hypertensive subjects, indicating a potential compensatory or protective role when iNOS is modulated.
Overall Impact on Oxidative/Nitrosative Stress	•eNOS uncoupling and iNOS overactivity contribute to superoxide generation and nitrosative stress, exacerbating myocardial remodeling in AH. •Interventions (BH_4_;, vitamin C) targeting these pathways help preserve endothelial function. NOS inhibition increases myocardial oxidative stress [174]. •eNOS uncoupling produces ROS [172]. •iNOS contributes to peroxynitrite formation, nitrosative stress, and endothelial dysfunction [177,184]. •iNOS deletion may improve function via reduced oxidative stress [180]. •iNOS upregulation promotes sympathoexcitation via oxidative stress [182].	•Hypertensive patients typically exhibit decreased NO bioavailability, increased free radical production, and heightened oxidative stress, all contributing to endothelial dysfunction and impaired vascular responses. Hypertensive individuals typically exhibit decreased NO, elevated free radicals, and increased oxidative stress [194]. •Increased oxidative stress reduces nNOS activity in hypertensive conditions [196,197].
Genetic and Biochemical Associations	•Animal models are primarily used to delineate mechanistic pathways (e.g., the effects of gene deletion or pharmacologic inhibition) rather than genetic polymorphisms.	•Genetic polymorphisms in the eNOS gene (e.g., Glu298Asp and intron 4a/b variants) are associated with essential hypertension (EH) and altered NO-mediated vasodilation. •Family history and genetic predisposition in humans further affect NO pathway efficiency and response to vasodilatory stimuli.

**Table 12:** Summary of current clinical practices alongside experimental strategies targeting multiple pathways—capillary density, fibrosis, mast cell activation, apelinergic signaling, VEGF/VEGFR modulation, and NO/NOS signaling in hypertensive heart disease.

Target/Pathway	Available Therapies	Experimental/Future Approaches
**Capillary Density (CD)**	• Chronic antihypertensive treatments (e.g., captopril) • Agents such as aspirin, methylprednisolone, and moxonidine (used post-infarction with observed improvements in capillary density)	• Strategies aimed at directly enhancing capillary regeneration and neovascularization (research focused on morphological evaluation as an efficacy indicator)
**Cardiac Fibrosis**	• RAAS inhibitors (e.g., losartan) that lower blood pressure and reduce ECM expansion • Anti-hypertensive drugs with secondary anti–TGF-β effects	• Colchicine (shown to reduce fibrosis in animal models) • MAPK/p38 inhibitors (with caution due to roles in scar integrity) • Calcium signaling modulators targeting channels (TRPC, Orai1) and calcineurin • Inhibitors of collagen cross-linking via LOX enzymes • Modulation of FGF signaling (FGF21 agonists, FGF23 antagonists, FGFR4 inhibitors) • Anti-inflammatory approaches (e.g., targeting MCP-1 or macrophage activity)
**Mast Cell Activation**	• Drugs targeting mast cell activation (currently in clinical trials for related disorders) • Histamine receptor antagonists (notably, H2 blockers have been associated with lower heart failure risk)	• Inhibitors of IgE-FcɛRI interactions • Chymase inhibitors (reduce ANG II formation and histamine release) • Tryptase inhibitors (potentially reduce fibroblast activation and fibrosis) • Anti-IgE therapies (e.g., omalizumab) targeting novel IgE–mast cell–IL-6 pathways • Anti-cytokine strategies addressing mast cell–derived IL-6, TNF-α, and TGF-β
**Apelin/APLNR System**	• Direct administration of native apelin peptides (e.g., [Pyr1]apelin-13) showing acute vasodilatory and antihypertensive effects • Exercise, which physiologically modulates apelin/APLNR expression	• Modified peptide analogues with enhanced stability to overcome short plasma half-life • Non-peptidic small molecule agonists (e.g., MM07, CMF-019, BMS-986224) • Development of biased agonists that selectively activate protective Gαi signaling while minimizing β-arrestin-mediated desensitization
**VEGF/VEGFR Pathway**	• Perindopril (shown to restore VEGF levels and support myocardial angiogenesis) • Trimetazidine (TMZ) promotes VEGF-A expression via Akt-HSF1-VEGF signaling • Exercise training enhances VEGF expression and NO bioavailability	• Combination therapeutic approaches that balance VEGF promotion with antihypertensive treatments to counteract the pro-hypertensive effects seen with VEGF inhibitors (VEGFIs) • Novel strategies to mitigate VEGFI-associated hypertension in cancer therapies
**NO/NOS Signaling**	• Established NO donors (organic nitrates such as isosorbide dinitrate; sodium nitroprusside for emergencies, L-arginine supplementation (improves endothelial NO production), Vitamin C (maintains BH_4_; bioavailability and prevents eNOS uncoupling) • Statins, ACE inhibitors, ARBs (which enhance eNOS activity and bioavailability, Lacidipine—calcium channel blocker reducing oxidative stress) • PDE5 inhibitors (sildenafil, tadalafil) and dietary nitrate/nitrite supplementation • sGC stimulators (e.g., riociguat) and activators (e.g., vericiguat)	• Novel NO delivery systems (e.g., biomaterial-based platforms, metal-nitrosyl complexes) • Cofactor supplementation strategies using BH4 or precursors like sepiapterin to restore eNOS coupling (limited by chemical instability) and sepiapterin (a more stable BH_4_; precursor via the salvage pathway. • Specific eNOS activators (e.g., AVE3085) • Isoform-selective modulators for nNOS and iNOS, aiming to harness protective effects without compromising other physiological roles; Nitrite administration; Selective iNOS inhibitors; Antioxidant therapies targeting NOS dysfunction • Advanced sGC activators that function independently of NO, and further refinement in modulating downstream cGMP signaling

## Data Availability

The raw data supporting the conclusions of this article will be made available by the authors upon request.

## Abbreviations

The following abbreviations are used in this manuscript:

AH	Arterial Hypertension
HHD	Hypertensive Heart Disease
LVH	Left Ventricular Hypertrophy
CD	Capillary Density
RAAS	Renin–Angiotensin–Aldosterone System
VEGF	Vascular Endothelial Growth Factor
VEGFR	Vascular Endothelial Growth Factor Receptor
NO	Nitric Oxide
NOS	Nitric Oxide Synthase
eNOS	Endothelial Nitric Oxide Synthase
iNOS	Inducible Nitric Oxide Synthase
MCN	Mast Cell Network
FGF-2	Fibroblast Growth Factor-2
ANG II	Angiotensin II
APLNR	Apelin Receptor
SHR	Spontaneously Hypertensive Rats
BH4	Tetrahydrobiopterin
2K1C	Two-Kidney, One-Clip (hypertension model)
LV	Left Ventricle
RV	Right Ventricle
L-NAME	N(G)-Nitro-L-arginine methyl ester (NOS inhibitor)
